# Experimental investigation and modelling of the mechanical properties of palm oil fuel ash concrete using Scheffe’s method

**DOI:** 10.1038/s41598-023-45987-3

**Published:** 2023-10-30

**Authors:** Godwin Adie Akeke, Philip-Edidiong Udo Inem, George Uwadiegwu Alaneme, Efiok Etim Nyah

**Affiliations:** 1 Department of Civil Engineering, University of Cross River State, Calabar, Nigeria; 2 Department of Civil Engineering, Kampala International University, Kampala, Uganda; 3 Department of Civil Engineering, Michael Okpara University of Agriculture, Umudike, Nigeria

**Keywords:** Materials science, Civil engineering

## Abstract

This study explores the enhancement of mechanical properties in concrete blended with palm oil fuel ash (POFA) through Scheffe's optimization. The utilization of POFA as supplementary cementitious material in concrete has gained attention for its potential environmental benefits. Utilizing a (5,2) simplex-lattice design, a systematic approach is employed for optimizing mixture proportions based on response parameters. The laboratory tests to evaluate concrete's mechanical behavior were conducted using the computed mixture ratios from the design experimental points after 28 days of hydration. The results showed maximum flexural strength at 8.84 N/mm^2^ and compressive strength at 31.16 N/mm^2^, achieved with a mix of 0.65:0.54:2.3:3.96:0.35 for cement, water, coarse aggregate, fine aggregate, and POFA. Additionally, maximum splitting tensile strength reached 8.84 N/mm^2^ with a mix of 0.62:0.55:2.09:3.86:0.38 for the same components. Conversely, the minimum flexural, splitting tensile and compressive strength within the experimental factor space was 4.25, 2.08 and 19.82 N/mm^2^ respectively. The results obtained indicated a satisfactory mechanical strength performance at POFA replacement of 35 percent in the concrete mixture. The developed mathematical model was statistically validated using analysis of variance (ANOVA) at a 95% confidence interval which showed satisfactory prediction performance. The findings from this study provide valuable insights into optimizing POFA-blended concrete for enhanced mechanical performance, offering potential sustainable solutions for the construction industry.

## Introduction

In concrete technology, the key goal is improving mechanical properties while embracing sustainability. Optimization techniques are pivotal for this purpose^1^. An innovative sustainable construction approach involves using agro waste ash in concrete, reducing environmental impact^2,3^. Agro waste, from agriculture, can make traditional concrete eco-friendlier and more efficient. This shift diverts agricultural residues from landfills and enhances concrete's performance and sustainability^4^. Ashes like rice husk, sugarcane bagasse, and palm oil fuel gain importance. Processed, they become valuable additives in concrete, improving workability, durability, and strength while reducing the carbon footprint^5,6^. This exploration delves into agro waste ash in concrete, from agricultural remnants to improving the built environment. Agro waste ash showcases sustainability and technology synergy, guiding us towards eco-conscious construction and a greener future^7^.

Scheffe mixture optimization is a systematic methodology used in various fields to enhance mixture performance, including materials science, engineering, and manufacturing^8^. It identifies optimal combinations of components within a mixture to achieve desired outcomes, considering interactions and component proportions^9,10^. In concrete, Scheffe optimization optimizes properties by adjusting proportions of cement, aggregates, and other materials^11^. Researchers and engineers can efficiently design mixtures, unlocking material potential and achieving desired performance with minimal experimentation^12^. The optimization of palm oil fuel ash (POFA) concrete using Scheffe's optimization approach has garnered considerable attention in recent literature. Researchers have explored this methodology to enhance various mechanical and durability properties of POFA concrete, contributing to the advancement of sustainable construction practices^13^. Hamada et al.^14^ investigated the use of response surface methodology to optimize the mechanical properties of palm oil clinker and nano-palm oil fuel ash blended concrete. Moreover, Mulizar et al.^15^ examined the engineering characteristics of geopolymer mortar derived from a blend of fly ash and POFA precursor. They achieved the highest compressive strength of 23 MPa when adding 5% POFA to the mix. The scanning electron microscopy analysis revealed that POFA particles have a coarse surface texture, which adversely affected workability and extended the setting time compared to fly ash-based geopolymer blends. Also, Onyia et al.^16^ carried out mathematical modeling of compressive strength in recycled ceramic tile aggregate concrete using a modified regression theory. The study explores the relationship between variables and compressive strength, offering insights into the properties of this sustainable construction material. Furthermore, The Okere et al.^17^ focuses on optimizing the cost of concrete mixes using Scheffe's simplex lattice theory. It explores efficient mix designs to minimize costs while maintaining quality. The research emphasized the role of Scheffe's optimization in aligning concrete design with sustainability goals. The literature on optimizing the mechanical properties of Palm Oil Fuel Ash (POFA) concrete using Scheffe's Method reveals several research gaps. These gaps include the limited focus on specific properties, variability in POFA characteristics, the influence of environmental factors, real-world project applications, and optimization under various constraints. Addressing these gaps is essential for advancing the understanding and practical application of Scheffe’s optimization in enhancing POFA concrete's mechanical properties while promoting sustainability in the construction industry.

This research explores palm oil fuel ash (POFA), a waste byproduct with potential in concrete applications, using Scheffe’s optimization—a statistical method. The goal is to determine the precise mix proportions and conditions that enhance POFA concrete’s mechanical strength. By leveraging Scheffe’s optimization, this study aims to elevate POFA concrete’s mechanical properties in line with the concrete industry’s shift toward sustainability^18,19^. Material proportions, mix design, and mechanical responses take center stage. POFA, a byproduct of the palm oil industry, holds promise for improving concrete properties and responsibly using industrial waste in a sustainable construction context^20^. Scheffe’s optimization is a statistical approach for systematically identifying the optimal ingredient proportions in concrete to achieve desired properties. It employs mathematical modeling and statistical analysis to discover the ideal combination of factors like cement, water, aggregates, and additives^21^. In contrast, the traditional method depends on practical experience and intuition, typically requiring multiple trial batches and adjustments, which can be a time-consuming process^22^. This study on Scheffe’s optimization of POFA concrete is highly relevant in sustainable construction and materials science. It employs Scheffe optimization to enhance mechanical performance while promoting eco-conscious practices^23^. The research aims to reveal POFA concrete’s untapped potential and establish a paradigm for optimizing concrete properties in line with sustainability. This journey seeks to reshape concrete's mechanical landscape and contribute to an ecologically balanced construction ecosystem. Ultimately, the focus on Scheffe’s optimization for POFA concrete aligns with contemporary construction demands, offering stronger, sustainable, and economically viable concrete solutions for our evolving world.

## Methodology

The methodology for this research study combines experimental design, mathematical modeling, and statistical validation to systematically optimize the mechanical properties of POFA concrete. Scheffe's mixture optimization is a robust methodology employed to optimize the composition of POFA concrete mixtures by determining the ideal proportions of their components^24,25^. Overall, Scheffe's mixture optimization is a valuable tool for efficiently and effectively optimizing mixture compositions, reducing trial and error, and enhancing material performance. Key steps in Scheffe's mixture optimization deployed in this study according to Attah et al*.*^26^:

1. Experimental Design: Designing a set of experiments with varying proportions of components in the mixture.
2. Response Variables: Identifying the properties or responses of interest that need optimization.
3. Data Collection: Conducting experiments and measuring the responses for each mixture.
4. Mathematical Modeling: Developing mathematical models to describe the relationships between component proportions and response variables.
5. Optimization: Using the model to find the optimal mixture proportions that yield desired response values.
6. Validation: Ensuring the optimized results are valid and reliable through additional tests or statistical analysis.

### Mathematical model development

Scheffe's concrete mixture optimization is a systematic methodology that employs Scheffe's principles to fine-tune the proportions of concrete constituents^27^. This approach is used to enhance specific properties of concrete, such as strength, durability, and workability. By systematically varying the mixture components within predefined ranges, engineers and researchers can determine the optimal combination that maximizes the desired properties while minimizing undesired effects^28,29^. Scheffe's optimization technique considers not only the individual contributions of each component but also their potential interactions. This comprehensive approach allows for a more accurate representation of how changes in component proportions affect the overall concrete performance^30^. Moreover, through mathematical modeling and statistical analysis, Scheffe's concrete mixture optimization helps to navigate the complex relationships among ingredients, ensuring the achievement of targeted concrete properties. This method offers a powerful tool for achieving high-quality concrete formulations that align with specific project requirements and sustainability goals^31^.

Scheffe's mixture model reveals a distinct correlation between component levels, impacting mixture uniformity. Changes in one component influence ingredient ratios for a consistent solution. Unlike regression models, it lacks an intercept term, assuring zero response with null factors. It adheres to a sum-to-one constraint and the total factor levels equate to unity, captured in Eqs. (1, 2) where x_i_ represents the fractions of mixture components and q is the total number of mixture ingredients^32^.

1 $$x_{1} + x_{2} + x_{3} + \cdots + x_{q - 1} + x_{q} = 1$$

2 $$\mathop \sum \limits_{i = 1}^{q} x_{i} = 1$$

The assessment of mixture response properties involves employing a mathematical (polynomial) function of degree m-order and q, where q represents the count of mixture components. This yields a (q, m) polynomial in the overall structure outlined in Eq. (3) and the parameter Y represents the response function^33^.

3 $$Y = b_{0} + \sum b_{i} x_{i} + \sum b_{ij} x_{i} x_{j} + \sum b_{ijk} x_{i} x_{j} x_{k} + \sum b_{i1,i2} \ldots i_{n} x_{i1} x_{i2} x_{im}$$

where $\left( {1 \le i \le q,1 \le i \le j \le q,1 \le i \le j \le k \le q} \right)$ and $b_{i}$ is the model coefficient

#### Scheffe’s Simplex lattice design

Scheffe's Simplex lattice design is a structured experimental approach used to optimize mixture compositions for various properties. It involves arranging experimental points in a systematic manner on a simplex lattice, where each point represents a specific mixture proportion. This design allows for the exploration of multiple factors simultaneously and accounts for their interactions^34,35^. The resulting data is used to create mathematical models that predict how changes in component proportions affect the desired outcomes^36^. A simplex is a geometrical shape with one more vertex than variable factor spaces (q), projected from n-dimensional space to n-1-dimensional coordinates. For q = 1, it's a two-vertex line; q = 2 forms a triangle, and q = 3 creates a tetrahedron^37^. Scheffe^28^ expanded and generalized the simplex lattice design, pioneering it in mixture design. His work established the notion of each component as a vertex within a regular simplex-lattice with q-1 factor space. Scheffe's simplex lattice patterns have become a widely recognized term for lattice designs, setting the stage for optimizing mixture compositions. Scheffe's Simplex lattice design is particularly useful in industries like materials science, where the properties of mixtures (e.g., in concrete or polymers) need to be optimized efficiently. It simplifies the experimentation process, reduces resource consumption, and provides insights into the complex relationships among mixture components^38^.

**Figure 1:**
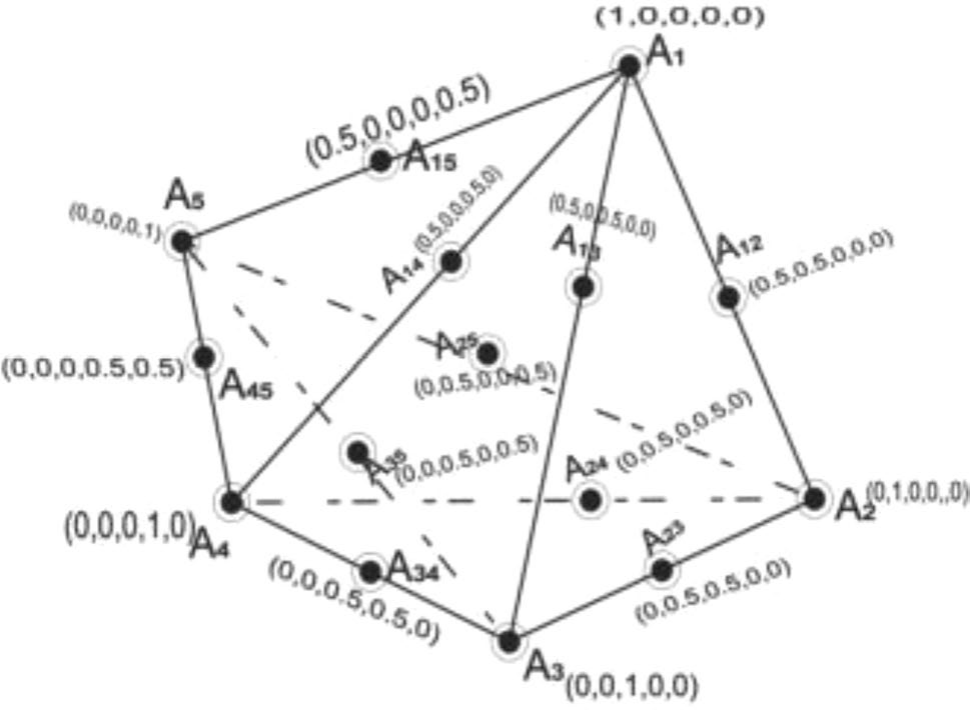
Scheffe’s simplex lattice structure with 15 experimental runs^48^.

A simplex design is a form of mixture design where design points are systematically positioned on a simplex lattice. The coordinate system employed for each ingredient's value $x_{i}$(i = 1, 2, …, q) is referred to as the simplex coordinate system. Here, q signifies the count of ingredients for each experimental run^45^. The simplex coordinate system is defined as $x_{i} = 0,\frac{1}{m},\frac{2}{m}, \ldots 1$. The design space encompasses all rational combinations of factor values, where m represents the lattice degree (or dimensional space). When considering an entire factor space in the design, a (q, m) simplex lattice emerges, characterized by uniformly distributed and saturated points^39^. Proportions assigned to each factor comprise m + 1 evenly spaced levels within the simplex coordinate system, with all feasible combinations stemming from these component concentration values. In the scenario of a quadratic lattice (q, 2), where the response surface is approximated by second-degree polynomials (m = 2), the simplex cooinate $x_{i} = 0,\frac{1}{2}, \ldots ,1$. is utilized, signifying 3 spaced levels. Similarly, in a full cubic mixture design model with m = 3, the simplex lattice coordinate $x_{i} = 0,\frac{1}{3},\frac{2}{3}, \ldots ,1$ indicates 4 spaced levels^40^. Every combination of these variables is applied in an experiment run, denoted as space points A_i_, A_ij_, or A_ijk_ for (i ≠ j ≠ k = 1, 2, 3, …, q). The summation of component volume fractions equates to one, and this constraint defines a regular tetrahedron in the simplex factor space. This arrangement is illustrated in Fig. 1.

#### Scheffe’s factor space and components interaction

Mixture experiment designs are arrangements for response surface experiments where each component adheres to a scale value between 0 and 1, with the sum of components equating to one. In a mixture containing q components, the shape and layout of experimental points are defined by q−1 component. These constraints establish a (q−1) dimensional design space known as a simplex. Essentially, the design space dimension is consistently one less than the component count (Borkowski et al., 2014). Consequently, the factor space manifests as a regular (q−1) dimensional simplex^41^.

In a (q−1) dimensional simplex, when q = 2, two connecting points create a straight-line simplex lattice. For q = 3, a triangular simplex lattice forms with equal sides; similarly, q = 4 yields a tetrahedron simplex lattice. (q−1) dimensions shape the boundary where q components interact within a mixture. The number of design space points N, represented as A_i_ or Aij or A_ijk_ for (i ≠ j ≠ k = 1, 2, 3, …, q), is determined by Eq. 3.4 in a simplex lattice design. This result guides the required number of experimental runs^42^.

4 $${\varvec{N}} = \frac{{\left( {{\varvec{q}} + {\varvec{m}} - 1} \right)!}}{{{\varvec{m}}!\left( {{\varvec{q}} - 1} \right)!}}$$

Thus, in the general canonical form of mixture models is mathematically expressed in Eqs. (5–7); for Linear, Second order/Quadratic and Cubic function respectively.

5 $$E\left( y \right) = \mathop \sum \limits_{i = 1}^{q} b_{i} x_{i}$$

6 $$E\left( y \right) = \mathop \sum \limits_{i = 1}^{q} b_{i} x_{i} + \sum \mathop \sum \limits_{i < j}^{q} b_{ij} x_{i} x_{j}$$

Substituting the values of *i* and *j* in Eq. (5) the quadratic equation for (0 ≤ i ≤ j ≤ 4) transforms to Eq. (7).

7 $${\varvec{E}}\left( {\varvec{y}} \right) = \mathop \sum \limits_{{{\varvec{i}} = 1}}^{{\varvec{q}}} {\varvec{b}}_{{\varvec{i}}} {\varvec{x}}_{{\varvec{i}}} + \sum \mathop \sum \limits_{{{\varvec{i}} < {\varvec{j}}}}^{{\varvec{q}}} {\varvec{b}}_{{{\varvec{ij}}}} {\varvec{x}}_{{\varvec{i}}} {\varvec{x}}_{{\varvec{j}}} + \sum \mathop \sum \limits_{{{\varvec{i}} < {\varvec{j}}}}^{{\varvec{q}}} {\varvec{b}}_{{{\varvec{ij}}}} {\varvec{x}}_{{\varvec{i}}} {\varvec{x}}_{{\varvec{j}}} \left( {{\varvec{x}}_{{\varvec{i}}} - {\varvec{x}}_{{\varvec{j}}} } \right) + \sum \sum \mathop \sum \limits_{{{\varvec{i}} < {\varvec{j}} < {\varvec{k}}}}^{{\varvec{q}}} {\varvec{b}}_{{{\varvec{ijk}}}} {\varvec{x}}_{{\varvec{i}}} {\varvec{x}}_{{\varvec{j}}} {\varvec{x}}_{{\varvec{k}}} \user2{ }$$

Here, $b_{i}$ represents the linear mixing proportion attributed to the pure blend when $x_{i}$ = 1 and $x_{j}$ = 0, where $i \ne j \ne k$. The expected response is denoted as $E\left( y \right)$. $b_{ij}$. signifies the quadratic non-linear blending between pairs of components, featuring parameters that could demonstrate either synergistic or antagonistic blending effects. Similarly, $b_{ijk}$ stands for the complete cun-linear blending coefficients among components, where parameters may indicate either synergistic or antagonistic blending interactions^43,44^.

#### Derivation of Scheffe’s quadratic response function

Further expansion of Eq. 6 through the substitution of $\left(0\le i\le j\le 5\right)$ into the values of *i* and *j* transforms to Eq. (8).

8 $$\begin{aligned} {\text{Y }} & = {\text{ b}}_{{\text{o}}} + {\text{ b}}_{{1}} {\text{X}}_{{1}} + {\text{ b}}_{{2}} {\text{X}}_{{2}} + {\text{ b}}_{{3}} {\text{X}}_{{3}} + {\text{ b}}_{{4}} {\text{X}}_{{4}} + {\text{ b}}_{{5}} {\text{X}}_{{5}} \\ & \;\; + {\text{ b}}_{{{11}}} {\text{X}}_{{1}}^{{2}} + {\text{ b}}_{{{12}}} {\text{X}}_{{1}} {\text{X}}_{{2}} + {\text{ b}}_{{{13}}} {\text{X}}_{{1}} {\text{X}}_{{3}} + {\text{ b}}_{{{14}}} {\text{X}}_{{1}} {\text{X}}_{{4}} + {\text{ b}}_{{{15}}} {\text{X}}_{{1}} {\text{X}}_{{5}} \\ & \;\; + {\text{ b}}_{{{22}}} {\text{X}}_{{2}}^{{2}} + {\text{ b}}_{{{23}}} {\text{X}}_{{2}} {\text{X}}_{{3}} + {\text{ b}}_{{{24}}} {\text{X}}_{{2}} {\text{X}}_{{4}} + {\text{ b}}_{{{25}}} {\text{X}}_{{2}} {\text{X}}_{{5}} \\ & \;\; + {\text{ b}}_{{{33}}} {\text{X}}_{{3}}^{{2}} + {\text{ b}}_{{{34}}} {\text{X}}_{{3}} {\text{X}}_{{4}} + {\text{ b}}_{{{35}}} {\text{X}}_{{3}} {\text{X}}_{{5}} + {\text{ b}}_{{{44}}} {\text{X}}_{{4}}^{{2}} + {\text{ b}}_{{{45}}} {\text{X}}_{{4}} {\text{X}}_{{5}} + {\text{ b}}_{{{55}}} {\text{X}}_{{5}}^{{2}} \\ \end{aligned}$$

Multiplying Eq. (1) by b_o_ we obtain a mathematical expression presented in Eq. (9)

9 $${\text{b}}_{0} = {\text{ b}}_{0} \left( {{\text{X}}_{{1}} + {\text{ X}}_{{2}} + {\text{ X}}_{{3}} + {\text{ X}}_{{4}} + {\text{ X}}_{{5}} } \right)$$

Multiplying in succession Eq. (2) by X_1_, X_2_, X_3_, X_4_, and X_5_ we get the relationship in Eq. (10)

10 $$\begin{gathered} {\text{X}}_{{1}}^{{2}} = {\text{ X}}_{{1}} {-}{\text{ X}}_{{1}} {\text{X}}_{{2}} - {\text{ X}}_{{1}} {\text{X}}_{{3}} - {\text{ X}}_{{1}} {\text{X}}_{{4}} - {\text{ X}}_{{1}} {\text{X}}_{{5}} \hfill \\ {\text{X}}_{{2}}^{{2}} = {\text{ X}}_{{2}} {-}{\text{ X}}_{{1}} {\text{X}}_{{2}} - {\text{ X}}_{{2}} {\text{X}}_{{3}} - {\text{ X}}_{{2}} {\text{X}}_{{4}} - {\text{ X}}_{{2}} {\text{X}}_{{5}} \hfill \\ {\text{X}}_{{3}}^{{2}} = {\text{ X}}_{{3}} - {\text{ X}}_{{1}} {\text{X}}_{{3}} - {\text{ X}}_{{2}} {\text{X}}_{{3}} - {\text{ X}}_{{3}} {\text{X}}_{{4}} - {\text{ X}}_{{3}} {\text{X}}_{{5}} \hfill \\ {\text{X}}_{{4}}^{{2}} = {\text{ X}}_{{4}} - {\text{ X}}_{{1}} {\text{X}}_{{4}} - {\text{ X}}_{{2}} {\text{X}}_{{4}} - {\text{ X}}_{{3}} {\text{X}}_{{4}} - {\text{ X}}_{{4}} {\text{X}}_{{5}} \hfill \\ {\text{X}}_{{5}}^{{2}} = {\text{ X}}_{{5}} {-}{\text{ X}}_{{1}} {\text{X}}_{{5}} - {\text{ X}}_{{2}} {\text{X}}_{{5}} - {\text{ X}}_{{3}} {\text{X}}_{{5}} - {\text{ X}}_{{4}} {\text{X}}_{{5}} \hfill \\ \end{gathered}$$

By inserting Eqs. (9 and 10) into Eq. (8), we derived the comprehensive quadratic polynomial model structure for a mixture consisting of five components, resulting in the expression given in Eq. (11).

11 $$\begin{aligned} {\hat{\text{Y}}} & = \, \left( {{\text{b}}_{0} + {\text{ b}}_{{1}} + {\text{ b}}_{{{11}}} } \right){\text{ X}}_{{1}} + \, \left( {{\text{b}}_{0} + {\text{ b}}_{{2}} + {\text{ b}}_{{{22}}} } \right){\text{ X}}_{{2}} + \, \left( {{\text{b}}_{0} + {\text{ b}}_{{3}} + {\text{ b}}_{{{33}}} } \right){\text{ X}}_{{3}} + \, \left( {{\text{b}}_{0} + {\text{ b}}_{{4}} + {\text{ b}}_{{{44}}} } \right){\text{ X}}_{{4}} \\ & \;\; + \, \left( {{\text{b}}_{0} + {\text{ b}}_{{5}} + {\text{ b}}_{{{55}}} } \right){\text{ X}}_{{5}} + \, \left( {{\text{b}}_{{{12}}} {-}{\text{ b}}_{{{11}}} - {\text{ b}}_{{{22}}} } \right){\text{ X}}_{{1}} {\text{X}}_{{2}} + \, \left( {{\text{b}}_{{{13}}} {-}{\text{ b}}_{{{11}}} - {\text{ b}}_{{{33}}} } \right){\text{ X}}_{{1}} {\text{X}}_{{3}} \\ & \;\; + \, \left( {{\text{b}}_{{{14}}} {-}{\text{ b}}_{{{11}}} - {\text{ b}}_{{{44}}} } \right){\text{ X}}_{{1}} {\text{X}}_{{4}} + \, \left( {{\text{b}}_{{{15}}} {-}{\text{ b}}_{{{11}}} - {\text{ b}}_{{{55}}} } \right){\text{ X}}_{{1}} {\text{X}}_{{5}} + \, \left( {{\text{b}}_{{{23}}} - {\text{ b}}_{{{22}}} - {\text{ b}}_{{{33}}} } \right){\text{ X}}_{{2}} {\text{X}}_{{3}} \\ & \;\; + \, \left( {{\text{b}}_{{{24}}} - {\text{ b}}_{{{22}}} - {\text{ b}}_{{{44}}} } \right){\text{ X}}_{{2}} {\text{X}}_{{4}} + \, \left( {{\text{b}}_{{{25}}} {-}{\text{ b}}_{{{11}}} {-\!\!-}{\text{b}}_{{{55}}} } \right){\text{ X}}_{{2}} {\text{X}}_{{5}} + \, \left( {{\text{b}}_{{{34}}} - {\text{ b}}_{{{33}}} - {\text{ b}}_{{{44}}} } \right){\text{ X}}_{{3}} {\text{X}}_{{4}} \\ & \;\; + \, \left( {{\text{b}}_{{{35}}} - {\text{ b}}_{{{33}}} - {\text{ b}}_{{{55}}} } \right){\text{ X}}_{{3}} {\text{X}}_{{5}} + \, \left( {{\text{b}}_{{{45}}} - {\text{ b}}_{{{44}}} - {\text{ b}}_{{{55}}} } \right){\text{ X}}_{{4}} {\text{X}}_{{5}} \\ \end{aligned}$$

We denote the mathematical relationship between the pure blends coefficients (β_i_) and the tenary blends (β_ij_) as shown in Eqs. (12, 13)

12 $$\beta_{{\text{i}}} = {\text{ b}}_{0} + {\text{ b}}_{{\text{i}}} + {\text{ b}}_{{{\text{ii}}}}$$

13 $$\beta_{{{\text{ij}}}} = {\text{ b}}_{{{\text{ij}}}} - {\text{ b}}_{{{\text{ii}}}} - {\text{ b}}_{{{\text{jj}}}}$$

Then we derive the second-degree polynomial for the response function as presented in Eq. (14).

14 $$\begin{aligned} E\left( y \right) & = \beta_{1} x_{1} + \beta_{2} x_{2} + \beta_{3} x_{3} + \beta_{4} x_{4} + \beta_{5} x_{5} + \beta_{12} x_{1} x_{2} \\ & \;\; + \beta_{13} x_{1} x_{3} + \beta_{14} x_{1} x_{4} + \beta_{15} x_{1} x_{5} + \beta_{23} x_{2} x_{3} + \beta_{24} x_{2} x_{4} \\ & \;\; + \beta_{25} x_{2} x_{5} + \beta_{34} x_{3} x_{4} + \beta_{35} x_{3} x_{5} + \beta_{45} x_{4} x_{5} \\ \end{aligned}$$

Here, ${x}_{i}$ signifies the coded variables known as pseudo components within the mixture design, whereas ${\beta }_{i}$ represents the response model coefficients in Scheffe's optimization equation. These coefficients can be denoted as ${\beta }_{i}$ for pure or binary blends, and as ${\beta }_{ij}$ for ternary blends or the amalgamation of mixture components. Their definition is formulated using the mathematical expression presented in Eq. (15)^45^.

15 $$\begin{aligned} \beta_{{{12}}} & = {\text{ 4Y}}_{{{12}}} {-}{\text{ 2Y}}_{{1}} {-}{\text{ 2Y}}_{{2}} ,\beta_{{{13}}} = {\text{ 4Y}}_{{{13}}} {-}{\text{ 2Y}}_{{1}} {-}{\text{ 2Y}}_{{3}} , \\ \beta_{{{14}}} & = {\text{ 4Y}}_{{{14}}} {-}{\text{ 2Y}}_{{1}} {-}{\text{ 2Y}}_{{4}} ,\beta_{{{23}}} = {\text{ 4Y}}_{{{23}}} {-}{\text{ 2Y}}_{{2}} {-}{\text{ 2Y}}_{{3}} , \\ \beta_{{{24}}} & = {\text{ 4Y}}_{{{24}}} {-}{\text{ 2Y}}_{{2}} {-}{\text{ 2Y}}_{{4}} ,\beta_{{{34}}} = {\text{ 4Y}}_{{{34}}} {-}{\text{ 2Y}}_{{3}} {-}{\text{ 2Y}}_{{4}} \\ \end{aligned}$$

Where $$\beta_{i} = Y_{i}$$ and $$\beta_{{{\text{ij}}}} = 4Y_{ij} - 2Y_{i} - 2Y_{j}$$.

#### Actual components and pseudo components

Pseudo-components are designated as fictitious or encoded variables adopted to streamline design creation and model adjustment. This approach curtails the linkages between component boundaries in constrained designs, resulting in reduced correlations among coefficients. This is accomplished by converting actual components, Z, into pseudo-components, X. Essentially, pseudo-components recalibrate the restricted data space to align with zero as the minimum permissible value (lower bound) for each component in mixture designs, similar to Scheffe's model^46^. This mathematical association is illustrated in Eq. (16).

16 $${\text{Z }} = {\text{ AX}}$$

Z signifies the real components which is the actual fraction of ingredients added to the mixture for a given run of experiment, whereas X stands for the surrogate components, with A as the constant, represented by a 5 × 5 matrix. The matrix A components are derived from the initial five mix ratios, forming a $q\times q$ dimensional matrix based on this preliminary experimental mixture^47^.

### Mix ratio development

The generation of initial trial mixes was initiated through a combination of expert judgment, practical experience, economic considerations, and insights from pertinent literature. This approach aimed to initiate the calculation of interaction points using Eq. (16) The aim is to find an optimal blend of mixture components that enhances desired properties. Scheffe’s methodology enables the identification of interactions and correlations between components, leading to a refined mix ratio that achieves enhanced performance in various applications such as concrete, materials, and other fields. By iteratively adjusting the mixture proportions and evaluating the predicted responses, the optimal mix ratio can be identified that maximizes or minimizes the desired properties^48^.

#### Mixture formulation computation

The initial mix ratios are:

**Z**_**1**_ [0.95**:**0.46**:**2.0**:**4.0**:**0.05], **Z**_**2**_ [0.85**:**0.46**:**1.85**:**3.75**:**0.15], **Z**_**3**_ [0.70**:**0.5**:**2.15**:**4.15**:**0.3], **Z**_**4**_ [0.65**:**0.55**:**2.25**:**3.92**:**0.35], **Z**_**5**_ [0.5**:**0.6**:**1.75**:**3.64**:**0.5].

The corresponding pseudo mix ratios are:

**X**_**1**_ [1:0:0:0:0], **X**_**2**_ [0:1:0:0:0], **X**_**3**_ [0:0:1:0:0], **X**_**4**_ [0:0:0:1:0], **X**_**5**_ [0:0:0:0:1].

Substitution of X_i_ and Z_i_ into Eq. (16) helps to calculate the pseudo components from the corresponding actual mixture components.

X_1_ = fraction of ordinary Portland cement; X_2_ = fraction of water cement ratio; X_3_ = fraction of fine aggregate.

X_4_ = fraction of coarse aggregate; X_5_ = fraction of POFA.

The matrix notation form of Eq. (16) which was utilized for computation of the components’ ratio for the concrete mixture.

$$\left( {\begin{array}{*{20}c} {{\text{Z}}_{1} } \\ {{\text{Z}}_{2} } \\ {\begin{array}{*{20}c} {{\text{Z}}_{3} } \\ {\begin{array}{*{20}c} {{\text{Z}}_{4} } \\ {{\text{Z}}_{5} } \\ \end{array} } \\ \end{array} } \\ \end{array} } \right) = \left( {\begin{array}{*{20}c} {a_{11} } \\ {{\text{a}}_{21} } \\ {\begin{array}{*{20}c} {{\text{a}}_{31} } \\ {\begin{array}{*{20}c} {{\text{a}}_{41} } \\ {{\text{a}}_{51} } \\ \end{array} } \\ \end{array} } \\ \end{array} \begin{array}{*{20}c} {{\text{a}}_{12} } \\ {{\text{a}}_{22} } \\ {\begin{array}{*{20}c} {{\text{a}}_{32} } \\ {\begin{array}{*{20}c} {{\text{a}}_{42} } \\ {{\text{a}}_{52} } \\ \end{array} } \\ \end{array} } \\ \end{array} \begin{array}{*{20}c} {{\text{a}}_{13} } \\ {{\text{a}}_{23} } \\ {\begin{array}{*{20}c} {{\text{a}}_{33} } \\ {\begin{array}{*{20}c} {{\text{a}}_{43} } \\ {{\text{a}}_{53} } \\ \end{array} } \\ \end{array} } \\ \end{array} \begin{array}{*{20}c} {{\text{a}}_{14} } \\ {{\text{a}}_{24} } \\ {\begin{array}{*{20}c} {{\text{a}}_{34} } \\ {\begin{array}{*{20}c} {{\text{a}}_{44} } \\ {{\text{a}}_{54} } \\ \end{array} } \\ \end{array} } \\ \end{array} \begin{array}{*{20}c} {{\text{a}}_{15} } \\ {{\text{a}}_{25} } \\ {\begin{array}{*{20}c} {{\text{a}}_{35} } \\ {\begin{array}{*{20}c} {{\text{a}}_{45} } \\ {{\text{a}}_{55} } \\ \end{array} } \\ \end{array} } \\ \end{array} } \right)\left( {\begin{array}{*{20}c} {{\text{X}}_{1} } \\ {{\text{X}}_{2} } \\ {\begin{array}{*{20}c} {{\text{X}}_{3} } \\ {\begin{array}{*{20}c} {{\text{X}}_{4} } \\ {{\text{X}}_{5} } \\ \end{array} } \\ \end{array} } \\ \end{array} } \right)$$

*For the first run*

$$\left(\begin{array}{c}0.95\\ 0.46\\ \begin{array}{c}4.0\\ \begin{array}{c}2.0\\ 0.05\end{array}\end{array}\end{array}\right)=\left(\begin{array}{c}{a}_{11}\\ {\mathrm{a}}_{21}\\ \begin{array}{c}{\mathrm{a}}_{31}\\ \begin{array}{c}{\mathrm{a}}_{41}\\ {\mathrm{a}}_{51}\end{array}\end{array}\end{array} \begin{array}{c}{\mathrm{a}}_{12}\\ {\mathrm{a}}_{22}\\ \begin{array}{c}{\mathrm{a}}_{32}\\ \begin{array}{c}{\mathrm{a}}_{42}\\ {\mathrm{a}}_{52}\end{array}\end{array}\end{array} \begin{array}{c}{\mathrm{a}}_{13}\\ {\mathrm{a}}_{23}\\ \begin{array}{c}{\mathrm{a}}_{33}\\ \begin{array}{c}{\mathrm{a}}_{43}\\ {\mathrm{a}}_{53}\end{array}\end{array}\end{array} \begin{array}{c}{\mathrm{a}}_{14}\\ {\mathrm{a}}_{24}\\ \begin{array}{c}{\mathrm{a}}_{34}\\ \begin{array}{c}{\mathrm{a}}_{44}\\ {\mathrm{a}}_{54}\end{array}\end{array}\end{array} \begin{array}{c}{\mathrm{a}}_{15}\\ {\mathrm{a}}_{25}\\ \begin{array}{c}{\mathrm{a}}_{35}\\ \begin{array}{c}{\mathrm{a}}_{45}\\ {\mathrm{a}}_{55}\end{array}\end{array}\end{array}\right)\left(\begin{array}{c}1\\ 0\\ \begin{array}{c}0\\ \begin{array}{c}0\\ 0\end{array}\end{array}\end{array}\right)$$

$${a}_{11}$$ = 0.97, $${a}_{21}$$= 0.44, $${a}_{31}$$= 2.18, $${a}_{41}$$= 3.85, $${a}_{51}$$=0.03

*For the second run*

$$\left(\begin{array}{c}0.85\\ 0.48\\ \begin{array}{c}3.75\\ \begin{array}{c}1.85\\ 0.15\end{array}\end{array}\end{array}\right)=\left(\begin{array}{c}{a}_{11}\\ {\mathrm{a}}_{21}\\ \begin{array}{c}{\mathrm{a}}_{31}\\ \begin{array}{c}{\mathrm{a}}_{41}\\ {\mathrm{a}}_{51}\end{array}\end{array}\end{array} \begin{array}{c}{\mathrm{a}}_{12}\\ {\mathrm{a}}_{22}\\ \begin{array}{c}{\mathrm{a}}_{32}\\ \begin{array}{c}{\mathrm{a}}_{42}\\ {\mathrm{a}}_{52}\end{array}\end{array}\end{array} \begin{array}{c}{\mathrm{a}}_{13}\\ {\mathrm{a}}_{23}\\ \begin{array}{c}{\mathrm{a}}_{33}\\ \begin{array}{c}{\mathrm{a}}_{43}\\ {\mathrm{a}}_{53}\end{array}\end{array}\end{array} \begin{array}{c}{\mathrm{a}}_{14}\\ {\mathrm{a}}_{24}\\ \begin{array}{c}{\mathrm{a}}_{34}\\ \begin{array}{c}{\mathrm{a}}_{44}\\ {\mathrm{a}}_{54}\end{array}\end{array}\end{array} \begin{array}{c}{\mathrm{a}}_{15}\\ {\mathrm{a}}_{25}\\ \begin{array}{c}{\mathrm{a}}_{35}\\ \begin{array}{c}{\mathrm{a}}_{45}\\ {\mathrm{a}}_{55}\end{array}\end{array}\end{array}\right)\left(\begin{array}{c}0\\ 1\\ \begin{array}{c}0\\ \begin{array}{c}0\\ 0\end{array}\end{array}\end{array}\right)$$

$${\mathrm{a}}_{12}$$ = 0.88, $${\mathrm{a}}_{22}$$ = 0.47, $${\mathrm{a}}_{32}$$= 1.89, $${\mathrm{a}}_{42}$$= 4.15, $${\mathrm{a}}_{52}$$ = 0.12

*For the third run*

$$\left(\begin{array}{c}0.70\\ 0.50\\ \begin{array}{c}4.15\\ \begin{array}{c}2.15\\ 0.3\end{array}\end{array}\end{array}\right)=\left(\begin{array}{c}{a}_{11}\\ {\mathrm{a}}_{21}\\ \begin{array}{c}{\mathrm{a}}_{31}\\ \begin{array}{c}{\mathrm{a}}_{41}\\ {\mathrm{a}}_{51}\end{array}\end{array}\end{array} \begin{array}{c}{\mathrm{a}}_{12}\\ {\mathrm{a}}_{22}\\ \begin{array}{c}{\mathrm{a}}_{32}\\ \begin{array}{c}{\mathrm{a}}_{42}\\ {\mathrm{a}}_{52}\end{array}\end{array}\end{array} \begin{array}{c}{\mathrm{a}}_{13}\\ {\mathrm{a}}_{23}\\ \begin{array}{c}{\mathrm{a}}_{33}\\ \begin{array}{c}{\mathrm{a}}_{43}\\ {\mathrm{a}}_{53}\end{array}\end{array}\end{array} \begin{array}{c}{\mathrm{a}}_{14}\\ {\mathrm{a}}_{24}\\ \begin{array}{c}{\mathrm{a}}_{34}\\ \begin{array}{c}{\mathrm{a}}_{44}\\ {\mathrm{a}}_{54}\end{array}\end{array}\end{array} \begin{array}{c}{\mathrm{a}}_{15}\\ {\mathrm{a}}_{25}\\ \begin{array}{c}{\mathrm{a}}_{35}\\ \begin{array}{c}{\mathrm{a}}_{45}\\ {\mathrm{a}}_{55}\end{array}\end{array}\end{array}\right)\left(\begin{array}{c}0\\ 0\\ \begin{array}{c}1\\ \begin{array}{c}0\\ 0\end{array}\end{array}\end{array}\right)$$

$${\mathrm{a}}_{13}$$ = 0.74, $${\mathrm{a}}_{23}$$ = 0.5, $${\mathrm{a}}_{33}$$ = 2.23, $${\mathrm{a}}_{43}$$ = 4.0, $${\mathrm{a}}_{53}$$ = 0.26

*For the fourth run*

$$\left(\begin{array}{c}0.65\\ 0.55\\ \begin{array}{c}3.92\\ \begin{array}{c}2.25\\ 0.35\end{array}\end{array}\end{array}\right)=\left(\begin{array}{c}{a}_{11}\\ {\mathrm{a}}_{21}\\ \begin{array}{c}{\mathrm{a}}_{31}\\ \begin{array}{c}{\mathrm{a}}_{41}\\ {\mathrm{a}}_{51}\end{array}\end{array}\end{array} \begin{array}{c}{\mathrm{a}}_{12}\\ {\mathrm{a}}_{22}\\ \begin{array}{c}{\mathrm{a}}_{32}\\ \begin{array}{c}{\mathrm{a}}_{42}\\ {\mathrm{a}}_{52}\end{array}\end{array}\end{array} \begin{array}{c}{\mathrm{a}}_{13}\\ {\mathrm{a}}_{23}\\ \begin{array}{c}{\mathrm{a}}_{33}\\ \begin{array}{c}{\mathrm{a}}_{43}\\ {\mathrm{a}}_{53}\end{array}\end{array}\end{array} \begin{array}{c}{\mathrm{a}}_{14}\\ {\mathrm{a}}_{24}\\ \begin{array}{c}{\mathrm{a}}_{34}\\ \begin{array}{c}{\mathrm{a}}_{44}\\ {\mathrm{a}}_{54}\end{array}\end{array}\end{array} \begin{array}{c}{\mathrm{a}}_{15}\\ {\mathrm{a}}_{25}\\ \begin{array}{c}{\mathrm{a}}_{35}\\ \begin{array}{c}{\mathrm{a}}_{45}\\ {\mathrm{a}}_{55}\end{array}\end{array}\end{array}\right)\left(\begin{array}{c}0\\ 0\\ \begin{array}{c}0\\ \begin{array}{c}1\\ 0\end{array}\end{array}\end{array}\right)$$

$${\mathrm{a}}_{14}$$ = 0.65, $${\mathrm{a}}_{24}$$ = 0.55, $${\mathrm{a}}_{34}$$ = 2.30, $${\mathrm{a}}_{44}$$ = 3.96, $${\mathrm{a}}_{54}$$ = 0.35

*For the fifth run*

$$\left(\begin{array}{c}0.5\\ 0.6\\ \begin{array}{c}3.64\\ \begin{array}{c}1.75\\ 0.5\end{array}\end{array}\end{array}\right)=\left(\begin{array}{c}{a}_{11}\\ {\mathrm{a}}_{21}\\ \begin{array}{c}{\mathrm{a}}_{31}\\ \begin{array}{c}{\mathrm{a}}_{41}\\ {\mathrm{a}}_{51}\end{array}\end{array}\end{array} \begin{array}{c}{\mathrm{a}}_{12}\\ {\mathrm{a}}_{22}\\ \begin{array}{c}{\mathrm{a}}_{32}\\ \begin{array}{c}{\mathrm{a}}_{42}\\ {\mathrm{a}}_{52}\end{array}\end{array}\end{array} \begin{array}{c}{\mathrm{a}}_{13}\\ {\mathrm{a}}_{23}\\ \begin{array}{c}{\mathrm{a}}_{33}\\ \begin{array}{c}{\mathrm{a}}_{43}\\ {\mathrm{a}}_{53}\end{array}\end{array}\end{array} \begin{array}{c}{\mathrm{a}}_{14}\\ {\mathrm{a}}_{24}\\ \begin{array}{c}{\mathrm{a}}_{34}\\ \begin{array}{c}{\mathrm{a}}_{44}\\ {\mathrm{a}}_{54}\end{array}\end{array}\end{array} \begin{array}{c}{\mathrm{a}}_{15}\\ {\mathrm{a}}_{25}\\ \begin{array}{c}{\mathrm{a}}_{35}\\ \begin{array}{c}{\mathrm{a}}_{45}\\ {\mathrm{a}}_{55}\end{array}\end{array}\end{array}\right)\left(\begin{array}{c}0\\ 0\\ \begin{array}{c}0\\ \begin{array}{c}0\\ 1\end{array}\end{array}\end{array}\right)$$

$${\mathrm{a}}_{15}$$ = 0.5, $${\mathrm{a}}_{25}$$ =$$0.6$$, $${\mathrm{a}}_{35}$$ =1.95, $${\mathrm{a}}_{45}$$ = 3.75, $${\mathrm{a}}_{55}$$ = 0.5

Substituting the values of the constants, we have [A] matrix.

$$\left(\begin{array}{c}0.97\\ 0.44\\ \begin{array}{c}2.18\\ \begin{array}{c}3.85\\ 0.03\end{array}\end{array}\end{array} \begin{array}{c}0.88\\ 0.47\\ \begin{array}{c}1.89\\ \begin{array}{c}4.15\\ 0.12\end{array}\end{array}\end{array} \begin{array}{c}0.74\\ 0.50\\ \begin{array}{c}2.23\\ \begin{array}{c}4.00\\ 0.26\end{array}\end{array}\end{array} \begin{array}{c}0.65\\ 0.54\\ \begin{array}{c}2.30\\ \begin{array}{c}3.96\\ 0.35\end{array}\end{array}\end{array} \begin{array}{c}0.5\\ 0.6\\ \begin{array}{c}1.95\\ \begin{array}{c}3.72\\ 0.5\end{array}\end{array}\end{array}\right)$$

The initial five points correspond to the vertices of the simplex factor space, while the remaining ten points situated within the simplex, known as interaction points, are determined by applying Eq. (16) through the following substitution process:

*Therefore, for A*_*12*_

$$\left(\begin{array}{c}{\mathrm{Z}}_{1}\\ {\mathrm{Z}}_{2}\\ \begin{array}{c}{\mathrm{Z}}_{3}\\ \begin{array}{c}{\mathrm{Z}}_{4}\\ {\mathrm{Z}}_{5}\end{array}\end{array}\end{array}\right)=\left(\begin{array}{c}0.97\\ 0.44\\ \begin{array}{c}2.18\\ \begin{array}{c}3.85\\ 0.03\end{array}\end{array}\end{array} \begin{array}{c}0.88\\ 0.47\\ \begin{array}{c}1.89\\ \begin{array}{c}4.15\\ 0.12\end{array}\end{array}\end{array} \begin{array}{c}0.74\\ 0.50\\ \begin{array}{c}2.23\\ \begin{array}{c}4.00\\ 0.26\end{array}\end{array}\end{array} \begin{array}{c}0.65\\ 0.54\\ \begin{array}{c}2.30\\ \begin{array}{c}3.96\\ 0.35\end{array}\end{array}\end{array} \begin{array}{c}0.5\\ 0.6\\ \begin{array}{c}1.95\\ \begin{array}{c}3.72\\ 0.5\end{array}\end{array}\end{array}\right)* \left(\begin{array}{c}0.5\\ 0.5\\ \begin{array}{c}0\\ \begin{array}{c}0\\ 0\end{array}\end{array}\end{array}\right)=\left(\begin{array}{c}0.925\\ 0.455\\ \begin{array}{c}2.035\\ \begin{array}{c}4\\ 0.075\end{array}\end{array}\end{array}\right)$$

*for A*_*13*_

$$\left(\begin{array}{c}{\mathrm{Z}}_{1}\\ {\mathrm{Z}}_{2}\\ \begin{array}{c}{\mathrm{Z}}_{3}\\ \begin{array}{c}{\mathrm{Z}}_{4}\\ {\mathrm{Z}}_{5}\end{array}\end{array}\end{array}\right)=\left(\begin{array}{c}0.97\\ 0.44\\ \begin{array}{c}2.18\\ \begin{array}{c}3.85\\ 0.03\end{array}\end{array}\end{array} \begin{array}{c}0.88\\ 0.47\\ \begin{array}{c}1.89\\ \begin{array}{c}4.15\\ 0.12\end{array}\end{array}\end{array} \begin{array}{c}0.74\\ 0.50\\ \begin{array}{c}2.23\\ \begin{array}{c}4.00\\ 0.26\end{array}\end{array}\end{array} \begin{array}{c}0.65\\ 0.54\\ \begin{array}{c}2.30\\ \begin{array}{c}3.96\\ 0.35\end{array}\end{array}\end{array} \begin{array}{c}0.5\\ 0.6\\ \begin{array}{c}1.95\\ \begin{array}{c}3.72\\ 0.5\end{array}\end{array}\end{array}\right)* \left(\begin{array}{c}0.5\\ 0\\ \begin{array}{c}0.5\\ \begin{array}{c}0\\ 0\end{array}\end{array}\end{array}\right)=\left(\begin{array}{c}0.855\\ 0.47\\ \begin{array}{c}2.205\\ \begin{array}{c}3.925\\ 0.145\end{array}\end{array}\end{array}\right)$$

*for A*_*14*_

$$\left(\begin{array}{c}{\mathrm{Z}}_{1}\\ {\mathrm{Z}}_{2}\\ \begin{array}{c}{\mathrm{Z}}_{3}\\ \begin{array}{c}{\mathrm{Z}}_{4}\\ {\mathrm{Z}}_{5}\end{array}\end{array}\end{array}\right)=\left(\begin{array}{c}0.97\\ 0.44\\ \begin{array}{c}2.18\\ \begin{array}{c}3.85\\ 0.03\end{array}\end{array}\end{array} \begin{array}{c}0.88\\ 0.47\\ \begin{array}{c}1.89\\ \begin{array}{c}4.15\\ 0.12\end{array}\end{array}\end{array} \begin{array}{c}0.74\\ 0.50\\ \begin{array}{c}2.23\\ \begin{array}{c}4.00\\ 0.26\end{array}\end{array}\end{array} \begin{array}{c}0.65\\ 0.54\\ \begin{array}{c}2.30\\ \begin{array}{c}3.96\\ 0.35\end{array}\end{array}\end{array} \begin{array}{c}0.5\\ 0.6\\ \begin{array}{c}1.95\\ \begin{array}{c}3.72\\ 0.5\end{array}\end{array}\end{array}\right)* \left(\begin{array}{c}0.5\\ 0\\ \begin{array}{c}0\\ \begin{array}{c}0.5\\ 0\end{array}\end{array}\end{array}\right)=\left(\begin{array}{c}0.81\\ 0.49\\ \begin{array}{c}2.24\\ \begin{array}{c}3.905\\ 0.19\end{array}\end{array}\end{array}\right)$$

*for A*_*15*_

$$\left(\begin{array}{c}{\mathrm{Z}}_{1}\\ {\mathrm{Z}}_{2}\\ \begin{array}{c}{\mathrm{Z}}_{3}\\ \begin{array}{c}{\mathrm{Z}}_{4}\\ {\mathrm{Z}}_{5}\end{array}\end{array}\end{array}\right)=\left(\begin{array}{c}0.97\\ 0.44\\ \begin{array}{c}2.18\\ \begin{array}{c}3.85\\ 0.03\end{array}\end{array}\end{array} \begin{array}{c}0.88\\ 0.47\\ \begin{array}{c}1.89\\ \begin{array}{c}4.15\\ 0.12\end{array}\end{array}\end{array} \begin{array}{c}0.74\\ 0.50\\ \begin{array}{c}2.23\\ \begin{array}{c}4.00\\ 0.26\end{array}\end{array}\end{array} \begin{array}{c}0.65\\ 0.54\\ \begin{array}{c}2.30\\ \begin{array}{c}3.96\\ 0.35\end{array}\end{array}\end{array} \begin{array}{c}0.5\\ 0.6\\ \begin{array}{c}1.95\\ \begin{array}{c}3.72\\ 0.5\end{array}\end{array}\end{array}\right)* \left(\begin{array}{c}0.5\\ 0\\ \begin{array}{c}0\\ \begin{array}{c}0\\ 0.5\end{array}\end{array}\end{array}\right)=\left(\begin{array}{c}0.735\\ 0.52\\ \begin{array}{c}2.065\\ \begin{array}{c}3.785\\ 0.265\end{array}\end{array}\end{array}\right)$$

*for A*_*23*_

$$\left(\begin{array}{c}{\mathrm{Z}}_{1}\\ {\mathrm{Z}}_{2}\\ \begin{array}{c}{\mathrm{Z}}_{3}\\ \begin{array}{c}{\mathrm{Z}}_{4}\\ {\mathrm{Z}}_{5}\end{array}\end{array}\end{array}\right)=\left(\begin{array}{c}0.97\\ 0.44\\ \begin{array}{c}2.18\\ \begin{array}{c}3.85\\ 0.03\end{array}\end{array}\end{array} \begin{array}{c}0.88\\ 0.47\\ \begin{array}{c}1.89\\ \begin{array}{c}4.15\\ 0.12\end{array}\end{array}\end{array} \begin{array}{c}0.74\\ 0.50\\ \begin{array}{c}2.23\\ \begin{array}{c}4.00\\ 0.26\end{array}\end{array}\end{array} \begin{array}{c}0.65\\ 0.54\\ \begin{array}{c}2.30\\ \begin{array}{c}3.96\\ 0.35\end{array}\end{array}\end{array} \begin{array}{c}0.5\\ 0.6\\ \begin{array}{c}1.95\\ \begin{array}{c}3.72\\ 0.5\end{array}\end{array}\end{array}\right)* \left(\begin{array}{c}0\\ 0.5\\ \begin{array}{c}0.5\\ \begin{array}{c}0\\ 0\end{array}\end{array}\end{array}\right)=\left(\begin{array}{c}0.81\\ 0.485\\ \begin{array}{c}2.06\\ \begin{array}{c}4.075\\ 0.19\end{array}\end{array}\end{array}\right)$$

*for A*_*24*_

$$\left(\begin{array}{c}{\mathrm{Z}}_{1}\\ {\mathrm{Z}}_{2}\\ \begin{array}{c}{\mathrm{Z}}_{3}\\ \begin{array}{c}{\mathrm{Z}}_{4}\\ {\mathrm{Z}}_{5}\end{array}\end{array}\end{array}\right)=\left(\begin{array}{c}0.97\\ 0.44\\ \begin{array}{c}2.18\\ \begin{array}{c}3.85\\ 0.03\end{array}\end{array}\end{array} \begin{array}{c}0.88\\ 0.47\\ \begin{array}{c}1.89\\ \begin{array}{c}4.15\\ 0.12\end{array}\end{array}\end{array} \begin{array}{c}0.74\\ 0.50\\ \begin{array}{c}2.23\\ \begin{array}{c}4.00\\ 0.26\end{array}\end{array}\end{array} \begin{array}{c}0.65\\ 0.54\\ \begin{array}{c}2.30\\ \begin{array}{c}3.96\\ 0.35\end{array}\end{array}\end{array} \begin{array}{c}0.5\\ 0.6\\ \begin{array}{c}1.95\\ \begin{array}{c}3.72\\ 0.5\end{array}\end{array}\end{array}\right)* \left(\begin{array}{c}0\\ 0.5\\ \begin{array}{c}0\\ \begin{array}{c}0.5\\ 0\end{array}\end{array}\end{array}\right)=\left(\begin{array}{c}0.765\\ 0.505\\ \begin{array}{c}2.095\\ \begin{array}{c}4.055\\ 0.235\end{array}\end{array}\end{array}\right)$$

*for A*_*25*_

$$\left(\begin{array}{c}{\mathrm{Z}}_{1}\\ {\mathrm{Z}}_{2}\\ \begin{array}{c}{\mathrm{Z}}_{3}\\ \begin{array}{c}{\mathrm{Z}}_{4}\\ {\mathrm{Z}}_{5}\end{array}\end{array}\end{array}\right)=\left(\begin{array}{c}0.97\\ 0.44\\ \begin{array}{c}2.18\\ \begin{array}{c}3.85\\ 0.03\end{array}\end{array}\end{array} \begin{array}{c}0.88\\ 0.47\\ \begin{array}{c}1.89\\ \begin{array}{c}4.15\\ 0.12\end{array}\end{array}\end{array} \begin{array}{c}0.74\\ 0.50\\ \begin{array}{c}2.23\\ \begin{array}{c}4.00\\ 0.26\end{array}\end{array}\end{array} \begin{array}{c}0.65\\ 0.54\\ \begin{array}{c}2.30\\ \begin{array}{c}3.96\\ 0.35\end{array}\end{array}\end{array} \begin{array}{c}0.5\\ 0.6\\ \begin{array}{c}1.95\\ \begin{array}{c}3.72\\ 0.5\end{array}\end{array}\end{array}\right)* \left(\begin{array}{c}0\\ 0.5\\ \begin{array}{c}0\\ \begin{array}{c}0\\ 0.5\end{array}\end{array}\end{array}\right)=\left(\begin{array}{c}0.69\\ 0.535\\ \begin{array}{c}1.92\\ \begin{array}{c}3.935\\ 0.31\end{array}\end{array}\end{array}\right)$$

*for A*_*34*_

$$\left(\begin{array}{c}{\mathrm{Z}}_{1}\\ {\mathrm{Z}}_{2}\\ \begin{array}{c}{\mathrm{Z}}_{3}\\ \begin{array}{c}{\mathrm{Z}}_{4}\\ {\mathrm{Z}}_{5}\end{array}\end{array}\end{array}\right)=\left(\begin{array}{c}0.97\\ 0.44\\ \begin{array}{c}2.18\\ \begin{array}{c}3.85\\ 0.03\end{array}\end{array}\end{array} \begin{array}{c}0.88\\ 0.47\\ \begin{array}{c}1.89\\ \begin{array}{c}4.15\\ 0.12\end{array}\end{array}\end{array} \begin{array}{c}0.74\\ 0.50\\ \begin{array}{c}2.23\\ \begin{array}{c}4.00\\ 0.26\end{array}\end{array}\end{array} \begin{array}{c}0.65\\ 0.54\\ \begin{array}{c}2.30\\ \begin{array}{c}3.96\\ 0.35\end{array}\end{array}\end{array} \begin{array}{c}0.5\\ 0.6\\ \begin{array}{c}1.95\\ \begin{array}{c}3.72\\ 0.5\end{array}\end{array}\end{array}\right)* \left(\begin{array}{c}0\\ 0\\ \begin{array}{c}0.5\\ \begin{array}{c}0.5\\ 0\end{array}\end{array}\end{array}\right)=\left(\begin{array}{c}0.695\\ 0.52\\ \begin{array}{c}2.265\\ \begin{array}{c}3.98\\ 0.305\end{array}\end{array}\end{array}\right)$$

*for A*_*35*_

$$\left(\begin{array}{c}{\mathrm{Z}}_{1}\\ {\mathrm{Z}}_{2}\\ \begin{array}{c}{\mathrm{Z}}_{3}\\ \begin{array}{c}{\mathrm{Z}}_{4}\\ {\mathrm{Z}}_{5}\end{array}\end{array}\end{array}\right)=\left(\begin{array}{c}0.97\\ 0.44\\ \begin{array}{c}2.18\\ \begin{array}{c}3.85\\ 0.03\end{array}\end{array}\end{array} \begin{array}{c}0.88\\ 0.47\\ \begin{array}{c}1.89\\ \begin{array}{c}4.15\\ 0.12\end{array}\end{array}\end{array} \begin{array}{c}0.74\\ 0.50\\ \begin{array}{c}2.23\\ \begin{array}{c}4.00\\ 0.26\end{array}\end{array}\end{array} \begin{array}{c}0.65\\ 0.54\\ \begin{array}{c}2.30\\ \begin{array}{c}3.96\\ 0.35\end{array}\end{array}\end{array} \begin{array}{c}0.5\\ 0.6\\ \begin{array}{c}1.95\\ \begin{array}{c}3.72\\ 0.5\end{array}\end{array}\end{array}\right)* \left(\begin{array}{c}0\\ 0\\ \begin{array}{c}0.5\\ \begin{array}{c}0\\ 0.5\end{array}\end{array}\end{array}\right)=\left(\begin{array}{c}0.62\\ 0.55\\ \begin{array}{c}2.09\\ \begin{array}{c}3.86\\ 0.38\end{array}\end{array}\end{array}\right)$$

*for A*_*45*_

$$\left(\begin{array}{c}{\mathrm{Z}}_{1}\\ {\mathrm{Z}}_{2}\\ \begin{array}{c}{\mathrm{Z}}_{3}\\ \begin{array}{c}{\mathrm{Z}}_{4}\\ {\mathrm{Z}}_{5}\end{array}\end{array}\end{array}\right)=\left(\begin{array}{c}0.97\\ 0.44\\ \begin{array}{c}2.18\\ \begin{array}{c}3.85\\ 0.03\end{array}\end{array}\end{array} \begin{array}{c}0.88\\ 0.47\\ \begin{array}{c}1.89\\ \begin{array}{c}4.15\\ 0.12\end{array}\end{array}\end{array} \begin{array}{c}0.74\\ 0.50\\ \begin{array}{c}2.23\\ \begin{array}{c}4.00\\ 0.26\end{array}\end{array}\end{array} \begin{array}{c}0.65\\ 0.54\\ \begin{array}{c}2.30\\ \begin{array}{c}3.96\\ 0.35\end{array}\end{array}\end{array} \begin{array}{c}0.5\\ 0.6\\ \begin{array}{c}1.95\\ \begin{array}{c}3.72\\ 0.5\end{array}\end{array}\end{array}\right)* \left(\begin{array}{c}0\\ 0\\ \begin{array}{c}0\\ \begin{array}{c}0.5\\ 0.5\end{array}\end{array}\end{array}\right)=\left(\begin{array}{c}0.575\\ 0.57\\ \begin{array}{c}2.125\\ \begin{array}{c}3.84\\ 0.425\end{array}\end{array}\end{array}\right)$$

The computation matrix table for the mixture proportion formulation is presented in Table 1.

**Table 1:** Second order mixture formulation matrix table.

Actual						Pseudo				
Z1	Z2	Z3	Z4	Z5	Response	X1	X2	X3	X4	X5
0.97	0.44	2.18	3.85	0.03	Y1	1	0	0	0	0
0.88	0.47	1.89	4.15	0.12	Y2	0	1	0	0	0
0.74	0.5	2.23	4	0.26	Y3	0	0	1	0	0
0.65	0.54	2.3	3.96	0.35	Y4	0	0	0	1	0
0.5	0.6	1.95	3.72	0.5	Y5	0	0	0	0	1
0.925	0.455	2.035	4	0.075	Y12	0.5	0.5	0	0	0
0.855	0.47	2.205	3.925	0.145	Y13	0.5	0	0.5	0	0
0.81	0.49	2.24	3.905	0.19	Y14	0.5	0	0	0.5	0
0.735	0.52	2.065	3.785	0.265	Y15	0.5	0	0	0	0.5
0.81	0.485	2.06	4.075	0.19	Y23	0	0.5	0.5	0	0
0.765	0.505	2.095	4.055	0.235	Y24	0	0.5	0	0.5	0
0.69	0.535	1.92	3.935	0.31	Y25	0	0.5	0	0	0.5
0.695	0.52	2.265	3.98	0.305	Y34	0	0	0.5	0.5	0
0.62	0.55	2.09	3.86	0.38	Y35	0	0	0.5	0	0.5
0.575	0.57	2.125	3.84	0.425	Y45	0	0	0	0.5	0.5

The mixture compositions for the experimental control points are also computed. These compositions were specifically designed to validate the generated Scheffe's regression model.

*for C*_*1*_

$$\left(\begin{array}{c}{\mathrm{Z}}_{1}\\ {\mathrm{Z}}_{2}\\ \begin{array}{c}{\mathrm{Z}}_{3}\\ \begin{array}{c}{\mathrm{Z}}_{4}\\ {\mathrm{Z}}_{5}\end{array}\end{array}\end{array}\right)=\left(\begin{array}{c}0.97\\ 0.44\\ \begin{array}{c}2.18\\ \begin{array}{c}3.85\\ 0.03\end{array}\end{array}\end{array} \begin{array}{c}0.88\\ 0.47\\ \begin{array}{c}1.89\\ \begin{array}{c}4.15\\ 0.12\end{array}\end{array}\end{array} \begin{array}{c}0.74\\ 0.50\\ \begin{array}{c}2.23\\ \begin{array}{c}4.00\\ 0.26\end{array}\end{array}\end{array} \begin{array}{c}0.65\\ 0.54\\ \begin{array}{c}2.30\\ \begin{array}{c}3.96\\ 0.35\end{array}\end{array}\end{array} \begin{array}{c}0.5\\ 0.6\\ \begin{array}{c}1.95\\ \begin{array}{c}3.72\\ 0.5\end{array}\end{array}\end{array}\right)* \left(\begin{array}{c}0.3\\ 0.3\\ \begin{array}{c}0.3\\ \begin{array}{c}0\\ 0.1\end{array}\end{array}\end{array}\right)=\left(\begin{array}{c}0.827\\ 0.483\\ \begin{array}{c}2.085\\ \begin{array}{c}3.972\\ 0.173\end{array}\end{array}\end{array}\right)$$

*for C*_*2*_

$$\left(\begin{array}{c}{\mathrm{Z}}_{1}\\ {\mathrm{Z}}_{2}\\ \begin{array}{c}{\mathrm{Z}}_{3}\\ \begin{array}{c}{\mathrm{Z}}_{4}\\ {\mathrm{Z}}_{5}\end{array}\end{array}\end{array}\right)=\left(\begin{array}{c}0.97\\ 0.44\\ \begin{array}{c}2.18\\ \begin{array}{c}3.85\\ 0.03\end{array}\end{array}\end{array} \begin{array}{c}0.88\\ 0.47\\ \begin{array}{c}1.89\\ \begin{array}{c}4.15\\ 0.12\end{array}\end{array}\end{array} \begin{array}{c}0.74\\ 0.50\\ \begin{array}{c}2.23\\ \begin{array}{c}4.00\\ 0.26\end{array}\end{array}\end{array} \begin{array}{c}0.65\\ 0.54\\ \begin{array}{c}2.30\\ \begin{array}{c}3.96\\ 0.35\end{array}\end{array}\end{array} \begin{array}{c}0.5\\ 0.6\\ \begin{array}{c}1.95\\ \begin{array}{c}3.72\\ 0.5\end{array}\end{array}\end{array}\right)* \left(\begin{array}{c}0.3\\ 0.3\\ \begin{array}{c}0\\ \begin{array}{c}0.3\\ 0.1\end{array}\end{array}\end{array}\right)=\left(\begin{array}{c}0.8\\ 0.495\\ \begin{array}{c}2.106\\ \begin{array}{c}3.96\\ 0.2\end{array}\end{array}\end{array}\right)$$

*for C*_*3*_

$$\left(\begin{array}{c}{\mathrm{Z}}_{1}\\ {\mathrm{Z}}_{2}\\ \begin{array}{c}{\mathrm{Z}}_{3}\\ \begin{array}{c}{\mathrm{Z}}_{4}\\ {\mathrm{Z}}_{5}\end{array}\end{array}\end{array}\right)=\left(\begin{array}{c}0.97\\ 0.44\\ \begin{array}{c}2.18\\ \begin{array}{c}3.85\\ 0.03\end{array}\end{array}\end{array} \begin{array}{c}0.88\\ 0.47\\ \begin{array}{c}1.89\\ \begin{array}{c}4.15\\ 0.12\end{array}\end{array}\end{array} \begin{array}{c}0.74\\ 0.50\\ \begin{array}{c}2.23\\ \begin{array}{c}4.00\\ 0.26\end{array}\end{array}\end{array} \begin{array}{c}0.65\\ 0.54\\ \begin{array}{c}2.30\\ \begin{array}{c}3.96\\ 0.35\end{array}\end{array}\end{array} \begin{array}{c}0.5\\ 0.6\\ \begin{array}{c}1.95\\ \begin{array}{c}3.72\\ 0.5\end{array}\end{array}\end{array}\right)* \left(\begin{array}{c}0.3\\ 0\\ \begin{array}{c}0.3\\ \begin{array}{c}0.3\\ 0.1\end{array}\end{array}\end{array}\right)=\left(\begin{array}{c}0.758\\ 0.504\\ \begin{array}{c}2.208\\ \begin{array}{c}3.915\\ 0.242\end{array}\end{array}\end{array}\right)$$

*for C*_*4*_

$$\left(\begin{array}{c}{\mathrm{Z}}_{1}\\ {\mathrm{Z}}_{2}\\ \begin{array}{c}{\mathrm{Z}}_{3}\\ \begin{array}{c}{\mathrm{Z}}_{4}\\ {\mathrm{Z}}_{5}\end{array}\end{array}\end{array}\right)=\left(\begin{array}{c}0.97\\ 0.44\\ \begin{array}{c}2.18\\ \begin{array}{c}3.85\\ 0.03\end{array}\end{array}\end{array} \begin{array}{c}0.88\\ 0.47\\ \begin{array}{c}1.89\\ \begin{array}{c}4.15\\ 0.12\end{array}\end{array}\end{array} \begin{array}{c}0.74\\ 0.50\\ \begin{array}{c}2.23\\ \begin{array}{c}4.00\\ 0.26\end{array}\end{array}\end{array} \begin{array}{c}0.65\\ 0.54\\ \begin{array}{c}2.30\\ \begin{array}{c}3.96\\ 0.35\end{array}\end{array}\end{array} \begin{array}{c}0.5\\ 0.6\\ \begin{array}{c}1.95\\ \begin{array}{c}3.72\\ 0.5\end{array}\end{array}\end{array}\right)* \left(\begin{array}{c}0\\ 0.3\\ \begin{array}{c}0.3\\ \begin{array}{c}0.3\\ 0.1\end{array}\end{array}\end{array}\right)=\left(\begin{array}{c}0.731\\ 0.513\\ \begin{array}{c}2.121\\ \begin{array}{c}4.005\\ 0.269\end{array}\end{array}\end{array}\right)$$

*for C*_*5*_

$$\left(\begin{array}{c}{\mathrm{Z}}_{1}\\ {\mathrm{Z}}_{2}\\ \begin{array}{c}{\mathrm{Z}}_{3}\\ \begin{array}{c}{\mathrm{Z}}_{4}\\ {\mathrm{Z}}_{5}\end{array}\end{array}\end{array}\right)=\left(\begin{array}{c}0.97\\ 0.44\\ \begin{array}{c}2.18\\ \begin{array}{c}3.85\\ 0.03\end{array}\end{array}\end{array} \begin{array}{c}0.88\\ 0.47\\ \begin{array}{c}1.89\\ \begin{array}{c}4.15\\ 0.12\end{array}\end{array}\end{array} \begin{array}{c}0.74\\ 0.50\\ \begin{array}{c}2.23\\ \begin{array}{c}4.00\\ 0.26\end{array}\end{array}\end{array} \begin{array}{c}0.65\\ 0.54\\ \begin{array}{c}2.30\\ \begin{array}{c}3.96\\ 0.35\end{array}\end{array}\end{array} \begin{array}{c}0.5\\ 0.6\\ \begin{array}{c}1.95\\ \begin{array}{c}3.72\\ 0.5\end{array}\end{array}\end{array}\right)* \left(\begin{array}{c}0.1\\ 0\\ \begin{array}{c}0.3\\ \begin{array}{c}0.3\\ 0.3\end{array}\end{array}\end{array}\right)=\left(\begin{array}{c}0.664\\ 0.536\\ \begin{array}{c}2.162\\ \begin{array}{c}3.889\\ 0.336\end{array}\end{array}\end{array}\right)$$

*for C*_*12*_

$$\left(\begin{array}{c}{\mathrm{Z}}_{1}\\ {\mathrm{Z}}_{2}\\ \begin{array}{c}{\mathrm{Z}}_{3}\\ \begin{array}{c}{\mathrm{Z}}_{4}\\ {\mathrm{Z}}_{5}\end{array}\end{array}\end{array}\right)=\left(\begin{array}{c}0.97\\ 0.44\\ \begin{array}{c}2.18\\ \begin{array}{c}3.85\\ 0.03\end{array}\end{array}\end{array} \begin{array}{c}0.88\\ 0.47\\ \begin{array}{c}1.89\\ \begin{array}{c}4.15\\ 0.12\end{array}\end{array}\end{array} \begin{array}{c}0.74\\ 0.50\\ \begin{array}{c}2.23\\ \begin{array}{c}4.00\\ 0.26\end{array}\end{array}\end{array} \begin{array}{c}0.65\\ 0.54\\ \begin{array}{c}2.30\\ \begin{array}{c}3.96\\ 0.35\end{array}\end{array}\end{array} \begin{array}{c}0.5\\ 0.6\\ \begin{array}{c}1.95\\ \begin{array}{c}3.72\\ 0.5\end{array}\end{array}\end{array}\right)* \left(\begin{array}{c}0.1\\ 0.3\\ \begin{array}{c}0\\ \begin{array}{c}0.3\\ 0.3\end{array}\end{array}\end{array}\right)=\left(\begin{array}{c}0.706\\ 0.527\\ \begin{array}{c}2.06\\ \begin{array}{c}3.934\\ 0.294\end{array}\end{array}\end{array}\right)$$

*for C*_*13*_

$$\left(\begin{array}{c}{\mathrm{Z}}_{1}\\ {\mathrm{Z}}_{2}\\ \begin{array}{c}{\mathrm{Z}}_{3}\\ \begin{array}{c}{\mathrm{Z}}_{4}\\ {\mathrm{Z}}_{5}\end{array}\end{array}\end{array}\right)=\left(\begin{array}{c}0.97\\ 0.44\\ \begin{array}{c}2.18\\ \begin{array}{c}3.85\\ 0.03\end{array}\end{array}\end{array} \begin{array}{c}0.88\\ 0.47\\ \begin{array}{c}1.89\\ \begin{array}{c}4.15\\ 0.12\end{array}\end{array}\end{array} \begin{array}{c}0.74\\ 0.50\\ \begin{array}{c}2.23\\ \begin{array}{c}4.00\\ 0.26\end{array}\end{array}\end{array} \begin{array}{c}0.65\\ 0.54\\ \begin{array}{c}2.30\\ \begin{array}{c}3.96\\ 0.35\end{array}\end{array}\end{array} \begin{array}{c}0.5\\ 0.6\\ \begin{array}{c}1.95\\ \begin{array}{c}3.72\\ 0.5\end{array}\end{array}\end{array}\right)* \left(\begin{array}{c}0.1\\ 0.3\\ \begin{array}{c}0.3\\ \begin{array}{c}0\\ 0.3\end{array}\end{array}\end{array}\right)=\left(\begin{array}{c}0.733\\ 0.515\\ \begin{array}{c}2.039\\ \begin{array}{c}3.946\\ 0.267\end{array}\end{array}\end{array}\right)$$

*for C*_*14*_

$$\left(\begin{array}{c}{\mathrm{Z}}_{1}\\ {\mathrm{Z}}_{2}\\ \begin{array}{c}{\mathrm{Z}}_{3}\\ \begin{array}{c}{\mathrm{Z}}_{4}\\ {\mathrm{Z}}_{5}\end{array}\end{array}\end{array}\right)=\left(\begin{array}{c}0.97\\ 0.44\\ \begin{array}{c}2.18\\ \begin{array}{c}3.85\\ 0.03\end{array}\end{array}\end{array} \begin{array}{c}0.88\\ 0.47\\ \begin{array}{c}1.89\\ \begin{array}{c}4.15\\ 0.12\end{array}\end{array}\end{array} \begin{array}{c}0.74\\ 0.50\\ \begin{array}{c}2.23\\ \begin{array}{c}4.00\\ 0.26\end{array}\end{array}\end{array} \begin{array}{c}0.65\\ 0.54\\ \begin{array}{c}2.30\\ \begin{array}{c}3.96\\ 0.35\end{array}\end{array}\end{array} \begin{array}{c}0.5\\ 0.6\\ \begin{array}{c}1.95\\ \begin{array}{c}3.72\\ 0.5\end{array}\end{array}\end{array}\right)* \left(\begin{array}{c}0.1\\ 0.3\\ \begin{array}{c}0.3\\ \begin{array}{c}0.3\\ 0\end{array}\end{array}\end{array}\right)=\left(\begin{array}{c}0.778\\ 0.497\\ \begin{array}{c}2.144\\ \begin{array}{c}4.018\\ 0.222\end{array}\end{array}\end{array}\right)$$

*for C*_*15*_

$$\left(\begin{array}{c}{\mathrm{Z}}_{1}\\ {\mathrm{Z}}_{2}\\ \begin{array}{c}{\mathrm{Z}}_{3}\\ \begin{array}{c}{\mathrm{Z}}_{4}\\ {\mathrm{Z}}_{5}\end{array}\end{array}\end{array}\right)=\left(\begin{array}{c}0.97\\ 0.44\\ \begin{array}{c}2.18\\ \begin{array}{c}3.85\\ 0.03\end{array}\end{array}\end{array} \begin{array}{c}0.88\\ 0.47\\ \begin{array}{c}1.89\\ \begin{array}{c}4.15\\ 0.12\end{array}\end{array}\end{array} \begin{array}{c}0.74\\ 0.50\\ \begin{array}{c}2.23\\ \begin{array}{c}4.00\\ 0.26\end{array}\end{array}\end{array} \begin{array}{c}0.65\\ 0.54\\ \begin{array}{c}2.30\\ \begin{array}{c}3.96\\ 0.35\end{array}\end{array}\end{array} \begin{array}{c}0.5\\ 0.6\\ \begin{array}{c}1.95\\ \begin{array}{c}3.72\\ 0.5\end{array}\end{array}\end{array}\right)* \left(\begin{array}{c}0.25\\ 0.25\\ \begin{array}{c}0.25\\ \begin{array}{c}0.25\\ 0\end{array}\end{array}\end{array}\right)=\left(\begin{array}{c}0.81\\ 0.4875\\ \begin{array}{c}2.15\\ \begin{array}{c}3.99\\ 0.19\end{array}\end{array}\end{array}\right)$$

*for C*_*23*_

$$\left(\begin{array}{c}{\mathrm{Z}}_{1}\\ {\mathrm{Z}}_{2}\\ \begin{array}{c}{\mathrm{Z}}_{3}\\ \begin{array}{c}{\mathrm{Z}}_{4}\\ {\mathrm{Z}}_{5}\end{array}\end{array}\end{array}\right)=\left(\begin{array}{c}0.97\\ 0.44\\ \begin{array}{c}2.18\\ \begin{array}{c}3.85\\ 0.03\end{array}\end{array}\end{array} \begin{array}{c}0.88\\ 0.47\\ \begin{array}{c}1.89\\ \begin{array}{c}4.15\\ 0.12\end{array}\end{array}\end{array} \begin{array}{c}0.74\\ 0.50\\ \begin{array}{c}2.23\\ \begin{array}{c}4.00\\ 0.26\end{array}\end{array}\end{array} \begin{array}{c}0.65\\ 0.54\\ \begin{array}{c}2.30\\ \begin{array}{c}3.96\\ 0.35\end{array}\end{array}\end{array} \begin{array}{c}0.5\\ 0.6\\ \begin{array}{c}1.95\\ \begin{array}{c}3.72\\ 0.5\end{array}\end{array}\end{array}\right)* \left(\begin{array}{c}0.25\\ 0.25\\ \begin{array}{c}0.25\\ \begin{array}{c}0\\ 0.25\end{array}\end{array}\end{array}\right)=\left(\begin{array}{c}0.7725\\ 0.525\\ \begin{array}{c}2.0625\\ \begin{array}{c}3.93\\ 0.2275\end{array}\end{array}\end{array}\right)$$

*for C*_*24*_

$$\left(\begin{array}{c}{\mathrm{Z}}_{1}\\ {\mathrm{Z}}_{2}\\ \begin{array}{c}{\mathrm{Z}}_{3}\\ \begin{array}{c}{\mathrm{Z}}_{4}\\ {\mathrm{Z}}_{5}\end{array}\end{array}\end{array}\right)=\left(\begin{array}{c}0.97\\ 0.44\\ \begin{array}{c}2.18\\ \begin{array}{c}3.85\\ 0.03\end{array}\end{array}\end{array} \begin{array}{c}0.88\\ 0.47\\ \begin{array}{c}1.89\\ \begin{array}{c}4.15\\ 0.12\end{array}\end{array}\end{array} \begin{array}{c}0.74\\ 0.50\\ \begin{array}{c}2.23\\ \begin{array}{c}4.00\\ 0.26\end{array}\end{array}\end{array} \begin{array}{c}0.65\\ 0.54\\ \begin{array}{c}2.30\\ \begin{array}{c}3.96\\ 0.35\end{array}\end{array}\end{array} \begin{array}{c}0.5\\ 0.6\\ \begin{array}{c}1.95\\ \begin{array}{c}3.72\\ 0.5\end{array}\end{array}\end{array}\right)* \left(\begin{array}{c}0.25\\ 0.25\\ \begin{array}{c}0\\ \begin{array}{c}0.25\\ 0.25\end{array}\end{array}\end{array}\right)=\left(\begin{array}{c}0.75\\ 0.5125\\ \begin{array}{c}2.08\\ \begin{array}{c}3.92\\ 0.25\end{array}\end{array}\end{array}\right)$$

*for C*_*25*_

$$\left(\begin{array}{c}{\mathrm{Z}}_{1}\\ {\mathrm{Z}}_{2}\\ \begin{array}{c}{\mathrm{Z}}_{3}\\ \begin{array}{c}{\mathrm{Z}}_{4}\\ {\mathrm{Z}}_{5}\end{array}\end{array}\end{array}\right)=\left(\begin{array}{c}0.97\\ 0.44\\ \begin{array}{c}2.18\\ \begin{array}{c}3.85\\ 0.03\end{array}\end{array}\end{array} \begin{array}{c}0.88\\ 0.47\\ \begin{array}{c}1.89\\ \begin{array}{c}4.15\\ 0.12\end{array}\end{array}\end{array} \begin{array}{c}0.74\\ 0.50\\ \begin{array}{c}2.23\\ \begin{array}{c}4.00\\ 0.26\end{array}\end{array}\end{array} \begin{array}{c}0.65\\ 0.54\\ \begin{array}{c}2.30\\ \begin{array}{c}3.96\\ 0.35\end{array}\end{array}\end{array} \begin{array}{c}0.5\\ 0.6\\ \begin{array}{c}1.95\\ \begin{array}{c}3.72\\ 0.5\end{array}\end{array}\end{array}\right)* \left(\begin{array}{c}0.25\\ 0\\ \begin{array}{c}0.25\\ \begin{array}{c}0.25\\ 0.25\end{array}\end{array}\end{array}\right)=\left(\begin{array}{c}0.715\\ 0.52\\ \begin{array}{c}2.165\\ \begin{array}{c}3.8825\\ 0.285\end{array}\end{array}\end{array}\right)$$

*for C*_*34*_

$$\left(\begin{array}{c}{\mathrm{Z}}_{1}\\ {\mathrm{Z}}_{2}\\ \begin{array}{c}{\mathrm{Z}}_{3}\\ \begin{array}{c}{\mathrm{Z}}_{4}\\ {\mathrm{Z}}_{5}\end{array}\end{array}\end{array}\right)=\left(\begin{array}{c}0.97\\ 0.44\\ \begin{array}{c}2.18\\ \begin{array}{c}3.85\\ 0.03\end{array}\end{array}\end{array} \begin{array}{c}0.88\\ 0.47\\ \begin{array}{c}1.89\\ \begin{array}{c}4.15\\ 0.12\end{array}\end{array}\end{array} \begin{array}{c}0.74\\ 0.50\\ \begin{array}{c}2.23\\ \begin{array}{c}4.00\\ 0.26\end{array}\end{array}\end{array} \begin{array}{c}0.65\\ 0.54\\ \begin{array}{c}2.30\\ \begin{array}{c}3.96\\ 0.35\end{array}\end{array}\end{array} \begin{array}{c}0.5\\ 0.6\\ \begin{array}{c}1.95\\ \begin{array}{c}3.72\\ 0.5\end{array}\end{array}\end{array}\right)* \left(\begin{array}{c}0\\ 0.25\\ \begin{array}{c}0.25\\ \begin{array}{c}0.25\\ 0.25\end{array}\end{array}\end{array}\right)=\left(\begin{array}{c}0.6925\\ 0.5275\\ \begin{array}{c}2.0925\\ \begin{array}{c}3.9575\\ 0.3075\end{array}\end{array}\end{array}\right)$$

*for C*_*35*_

$$\left(\begin{array}{c}{\mathrm{Z}}_{1}\\ {\mathrm{Z}}_{2}\\ \begin{array}{c}{\mathrm{Z}}_{3}\\ \begin{array}{c}{\mathrm{Z}}_{4}\\ {\mathrm{Z}}_{5}\end{array}\end{array}\end{array}\right)=\left(\begin{array}{c}0.97\\ 0.44\\ \begin{array}{c}2.18\\ \begin{array}{c}3.85\\ 0.03\end{array}\end{array}\end{array} \begin{array}{c}0.88\\ 0.47\\ \begin{array}{c}1.89\\ \begin{array}{c}4.15\\ 0.12\end{array}\end{array}\end{array} \begin{array}{c}0.74\\ 0.50\\ \begin{array}{c}2.23\\ \begin{array}{c}4.00\\ 0.26\end{array}\end{array}\end{array} \begin{array}{c}0.65\\ 0.54\\ \begin{array}{c}2.30\\ \begin{array}{c}3.96\\ 0.35\end{array}\end{array}\end{array} \begin{array}{c}0.5\\ 0.6\\ \begin{array}{c}1.95\\ \begin{array}{c}3.72\\ 0.5\end{array}\end{array}\end{array}\right)* \left(\begin{array}{c}0.2\\ 0.2\\ \begin{array}{c}0.2\\ \begin{array}{c}0.2\\ 0.2\end{array}\end{array}\end{array}\right)=\left(\begin{array}{c}0.748\\ 0.51\\ \begin{array}{c}2.11\\ \begin{array}{c}3.936\\ 0.252\end{array}\end{array}\end{array}\right)$$

*for C*_*45*_

$$\left(\begin{array}{c}{\mathrm{Z}}_{1}\\ {\mathrm{Z}}_{2}\\ \begin{array}{c}{\mathrm{Z}}_{3}\\ \begin{array}{c}{\mathrm{Z}}_{4}\\ {\mathrm{Z}}_{5}\end{array}\end{array}\end{array}\right)=\left(\begin{array}{c}0.97\\ 0.44\\ \begin{array}{c}2.18\\ \begin{array}{c}3.85\\ 0.03\end{array}\end{array}\end{array} \begin{array}{c}0.88\\ 0.47\\ \begin{array}{c}1.89\\ \begin{array}{c}4.15\\ 0.12\end{array}\end{array}\end{array} \begin{array}{c}0.74\\ 0.50\\ \begin{array}{c}2.23\\ \begin{array}{c}4.00\\ 0.26\end{array}\end{array}\end{array} \begin{array}{c}0.65\\ 0.54\\ \begin{array}{c}2.30\\ \begin{array}{c}3.96\\ 0.35\end{array}\end{array}\end{array} \begin{array}{c}0.5\\ 0.6\\ \begin{array}{c}1.95\\ \begin{array}{c}3.72\\ 0.5\end{array}\end{array}\end{array}\right)* \left(\begin{array}{c}0.3\\ 0.3\\ \begin{array}{c}0.3\\ \begin{array}{c}0.1\\ 0\end{array}\end{array}\end{array}\right)=\left(\begin{array}{c}0.842\\ 0.477\\ \begin{array}{c}2.12\\ \begin{array}{c}3.96\\ 0.158\end{array}\end{array}\end{array}\right)$$

The computation matrix table for the mixture proportion formulation is presented in Table 2.

**Table 2:** Second order mixture formulation matrix table for control points.

Actual						Pseudo				
Z1	Z2	Z3	Z4	Z5	Response	X1	X2	X3	X4	X5
0.827	0.483	2.085	3.972	0.173	C1	0.3	0.3	0.3	0	0.1
0.8	0.495	2.106	3.96	0.2	C2	0.3	0.3	0	0.3	0.1
0.758	0.504	2.208	3.915	0.242	C3	0.3	0	0.3	0.3	0.1
0.731	0.513	2.121	4.005	0.269	C4	0	0.3	0.3	0.3	0.1
0.664	0.536	2.162	3.889	0.336	C5	0.1	0	0.3	0.3	0.3
0.706	0.527	2.06	3.934	0.294	C12	0.1	0.3	0	0.3	0.3
0.733	0.515	2.039	3.946	0.267	C13	0.1	0.3	0.3	0	0.3
0.778	0.497	2.144	4.018	0.222	C14	0.1	0.3	0.3	0.3	0
0.81	0.4875	2.15	3.99	0.19	C15	0.25	0.25	0.25	0.25	0
0.7725	0.5025	2.0625	3.93	0.2275	C23	0.25	0.25	0.25	0	0.25
0.75	0.5125	2.08	3.92	0.25	C24	0.25	0.25	0	0.25	0.25
0.715	0.52	2.165	3.8825	0.285	C25	0.25	0	0.25	0.25	0.25
0.6925	0.5275	2.0925	3.9575	0.3075	C34	0	0.25	0.25	0.25	0.25
0.748	0.51	2.11	3.936	0.252	C35	0.2	0.2	0.2	0.2	0.2
0.842	0.477	2.12	3.996	0.158	C45	0.3	0.3	0.3	0.1	0

#### Test of adequacy of the model

The effectiveness of the developed Scheffe's model is evaluated using statistical techniques, specifically through analysis of variance (ANOVA). These tests are applied to ascertain any disparities between the actual outcomes and the model-predicted results for the control experiment^49^. The statistical assessment is performed separately for both second and third order models, with a 95% confidence level. It focuses on the achieved compressive and flexural strengths results. To derive the predicted values (Y-predicted) for the control test points, the model equation is employed by substituting the respective values of coded units X_1_, X_2_, X_3_, X_4_, and X_5_^50,51^.

•**Null Hypothesis**

There is no noteworthy distinction observed between the strength outcomes predicted by the model and those obtained from laboratory tests.

•**Alternative Hypothesis**

A considerable contrast exists between the strength outcomes projected by the model and those derived from laboratory testing.

### Materials

#### Palm oil fuel ash

POFA samples shall be collected in a sack from different location within Cross River South and Central Palm Mill. Global Positioning System (GPS) coordinates and site photographs shall also be taken. The collected POFA samples shall be taken to the laboratory where it is oven dried. Sieving of the POFA samples may be necessary to remove all unwanted materials contained in the sample in agreement with BS 8615-1 (2019) and ASTM C618 standards^51^.

#### Coarse aggregates

The coarse aggregates shall be 15–22 mm grain size, obtained from Saturn quarry at Akamkpa Local Government Area of the State.

#### Fine aggregates

Fine aggregates were obtained from the Calabar river and classified within the sharp sand envelope in accordance with BS EN12620 specifications.

#### Ordinary Portland cement

Material shall be obtained from United Cement Company of Nigeria (UNICEM), Akamkpa Local Government Area with 32.5 grade and normal consistency of 30% in accordance with Nigerian Industrial Standard (NIS) 444-1 requirement^52^.

#### Water

Drinkable or potable water, a crucial element, influences the mechanical and rheological characteristics of concrete, meeting the specifications outlined in ASTM C1602-12.

### Laboratory methods

#### Study area

**Figure 2:**
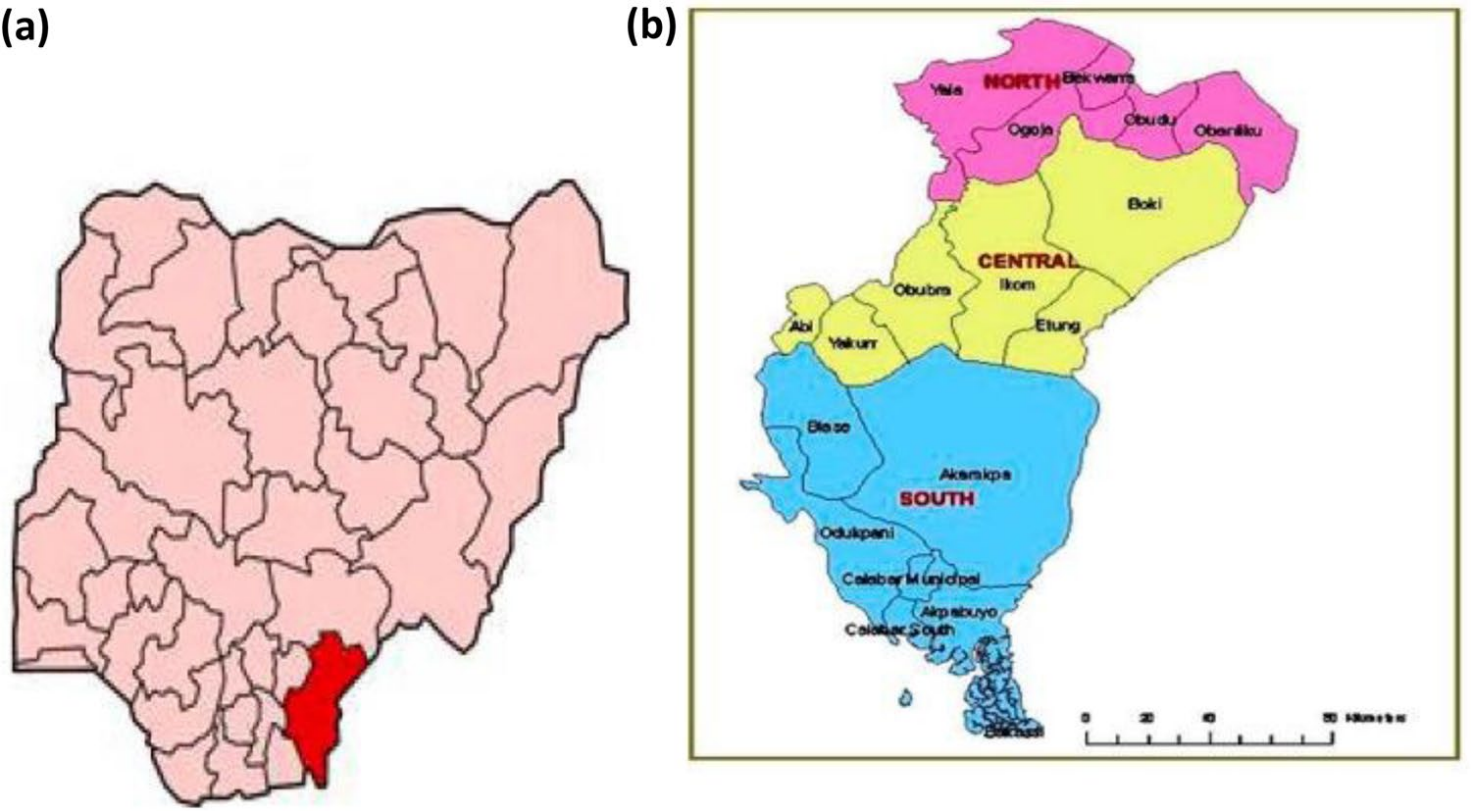
(**a**) Nigerian map showing Cross River State. (**b**) Cross River State showing various L.G.A.

The area of study under review was tailored around the availability of the concept material in view which is central and southern Cross River State shown in Fig. 2. Materials for the study will be obtained from Calabar Municipal, Ikom and Biase Local Government Areas. The laboratory work was done in the Concrete Laboratory of the Cross River University of Technology (CRUTECH), Calabar.

#### Experimental investigation and setup

The concrete mix involves partially substituting cement in the matrix with POFA. Utilizing Scheffe's statistical technique, the mix formulation was determined using the mathematical relationship between real and pseudo-components. The target strength of 25 N/mm^2^ was aimed for, with a cement content of 290 kg/m^3^, coarse aggregate content of 1198.65 kg/m^3^, and fine aggregate content of 766.35 kg/m^3^ for the design^53^. The concrete ingredients were mixed thoroughly with water to ensure uniformity before compaction and placement into the mold for mechanical strength assessments. Fresh concrete was also tested to evaluate setting time and workability. Following this, the concrete samples were cured in a tank at room temperature for 28 days after which they were subjected to structural property investigations. This study aims to explore the structural attributes of POFA concrete^54^.

#### Grain size distribution

Sieve analysis is a fundamental technique used to determine the grain size distribution of aggregates in concrete. This method involves passing a sample of aggregates through a series of sieves with progressively smaller openings in accordance with BS EN 933-1:2012 specification. The amount of material retained on each sieve is measured and used to create a particle size distribution curve. This curve provides valuable insights into the composition and grading of aggregates, which directly influence the properties of concrete, such as workability, strength, and durability. By analyzing the distribution of different particle sizes, engineers and researchers can optimize concrete mix designs, ensuring the right balance of coarse and fine aggregates for desired performance characteristics^55^.

#### Slump tests

The slump test is a commonly employed method to assess the workability or cohesion of newly mixed concrete. This technique offers insights into the concrete's capacity to flow and adapt without separation or excessive liquid release. Furthermore, it offers valuable insights into how effortlessly the concrete can be positioned, condensed, and refined during the construction process. Fresh concrete's workability is assessed through slump tests. These tests adhere to the guidelines stipulated in BS EN 12350-2:2009^56^.

#### Compressive strength

The compressive strength experiment is a fundamental test conducted on concrete specimens to assess their ability to withstand axial loads without failure. This procedure will be conducted on solidified concrete cubes with dimensions of 150mm x 150mm x 150mm, following the guidelines outlined in BS EN 12390-3:2019 for concrete compressive strength testing. After casting, the concrete cubes will be allowed to solidify for 24 h, followed by a 28-day curing period before testing. Subsequently, each cured sample will be placed between two plates in a Universal Test Machine (UTM), which will apply a load uniformly across the two opposing faces. The load will aim to flatten the sample, causing compression along the load's direction and expansion perpendicular to it. The loading will be incrementally increased at a rate of 140 kg/cm^2^ per minute until failure occurs. The compressive strength of each cube will then be calculated as the load at failure divided by the sample's surface area, expressed mathematically as shown in Eq. (17)^57,58^.

17 $$\sigma = \frac{F}{A}$$

where F is the applied load in (N) and A the cross-sectional area in (mm^2^)

#### Split tensile test

The splitting tensile strength experiment is a test conducted on cylindrical concrete specimens to evaluate their resistance to cracking and failure under tensile stresses. The cylindrical specimens are prepared and cured for a specific duration, commonly 28 days. After curing, the specimens are placed horizontally in a testing machine, and a diametrical load is applied to the cylindrical surface following the specifications defined in BS EN 12390-6:2019. The blended concrete mixture is filled into cylindrical molds measuring 100mm in diameter and 300mm in height. Following a day's period, the specimens are extracted from the molds and transferred to a water bath maintained at a consistent temperature range of 23–25 °C. This step is undertaken to determine the tensile strength of the mixture after it has been cured for 28  days, utilizing a crushing apparatus. The splitting tensile strength of the specimen is calculated using the formula presented in Eq. (18)^59,60^

18 $${\text{T }} = {\text{ 2P}}/\pi {\text{LD}}$$

where, T = Splitting tensile strength; P = Maximum applied load; L = Length, D = Diameter.

#### Flexural strength

The flexural strength experiment involves subjecting concrete beams or prisms to a bending load to evaluate their ability to withstand applied forces. This test measures the concrete's ability to resist bending stresses and is crucial for assessing its performance in structural applications. Concrete specimens, typically shaped like beams or prisms, are cast and cured according to standard procedures. After the curing period, these specimens are placed on a testing apparatus where a gradually increasing load is applied at the center of the specimen. As the load increases, the specimen bends until it eventually cracks and fails. The cement-POFA blended concrete were filled in a beam mold of 100 mm*100 mm*400 mm dimension, after 24 h the hardened samples are immersed in a curing tank and cured for 28 days in accordance with BS EN 12390-5:2019 requirement. The flexural strength response expressed in units of force per unit area, such as N/mm^2^ is determined using the mathematical expression in Eq. (19)^61^.

19 $$F \cdot S = \frac{P*L}{{2 *B*D^{2} }}$$

Here, P is the applied load at fracture; L is the beam’s length; B is the width and D is the beam’s thickness.

## Results discussion and analysis

The results, discussion, and analysis of the Scheffe’s optimization of the mechanical properties of palm oil fuel ash (POFA) concrete reveal significant insights into the behavior and performance of the optimized concrete mixture. Through the utilization of Scheffe's statistical approach, the study aimed to enhance the mechanical properties of concrete by incorporating POFA as a partial replacement for cement. The investigation began with the development of mixture ratios using mathematical models based on Scheffe’s approach. The desired characteristic strength of 25 N/mm^2^ was chosen, and the corresponding proportions of cement, coarse aggregate, POFA and fine aggregate were determined. The concrete mixture was prepared by thoroughly blending these ingredients with water to ensure homogeneity^62,63^.

### Characterization of test materials

**Figure 3:**
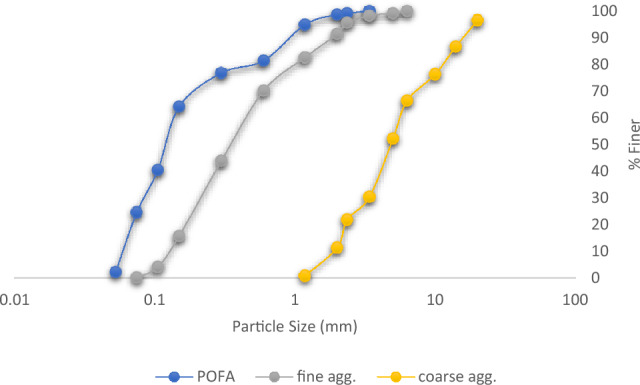
Particles size distribution plot.

Laboratory tests were conducted on the mixture components to assess their overall engineering behaviour as civil construction materials. The test aggregates and admixtures underwent sieve analysis and specific gravity tests to evaluate their gradation and particle size distribution. Figure 3 displays the sieve analysis test outcome represented on a semi-log graph, depicting the variation of soil grain sizes using a cumulative frequency distribution curve. The results indicate that for the coarse aggregate, the percentage passing through the sieve sizes 10-2 mm ranges from 75.3 to 11.5%. Similarly, for the fine aggregates and POFA, the percentage passing through sieve sizes 2 mm–75 μm ranges from 91.4 to 0.15% and 98.67–24.55%, respectively. The coefficients of gradation are provided in Table 3, showing well-graded sand and gravel particles that meet the specified requirements by BS 882, ensuring improved concrete durability performance^64,65^.

**Table 3:** Gradation coefficients.

Test materials	D_10_	D_30_	D_60_	C_u_	C_c_
Coarse Agg	1.9	3.4	6	3.158	1.014
POFA	0.06	0.088	0.14	2.333	0.922
Fine Agg	0.13	0.2	0.55	4.231	0.559

#### Chemical properties of the test cement and POFA

Table 4 presents the results of the chemical properties assessment of the test admixtures achieved using x-ray fluorescence (XRF). The findings show that POFA predominantly consists of SiO_2_ (62.8%), Al_2_O_3_ (4.6%), and Fe_2_O_3_ (3.02%), totaling 70.52% by composition indicating good pozzolanic properties in line with ASTM C618, 98 specifications. The test Portland cement and POFA under study from the obtained results, exhibit significant levels of calcium oxide (CaO) at 10.23% and 7.6% respectively. This abundance of CaO boosts complete hydration of cement, improving the mechanical strength and durability characteristics, as depicted in Table 4. The cement hydration reaction mechanism involves aluminum and silicon oxides derived from the pozzolanic material (POFA) blending with lime to form hydration products such as calcium silicate hydrate (C–S–H), resulting in a progressively harder mass over time^66,67^.

**Table 4:** Elemental composition of test samples using X-ray fluorescence (XRF).

Elemental Oxide	POFA (%)	Cement (%)
CaO	7.6	10.23
MgO	3.9	0.085
Fe_2_O_3_	3.02	6.11
Na_2_O	0.15	2.03
Al_2_O_3_	4.6	20.6
SiO_2_	62.8	51.9
SO_3_	0.24	0.13
LOI	9.42	3.62
TiO_2_	Trace	0.55
K_2_O	Trace	2.47

### Test on fresh concrete specimen

#### Slump test results

**Figure 4:**
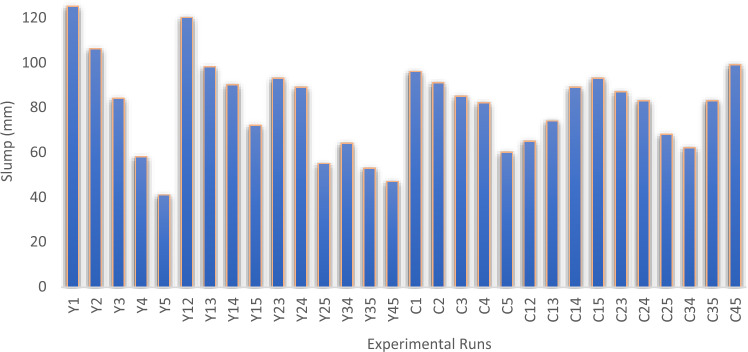
Slump result.

Laboratory tests were conducted to assess the workability properties of the freshly mixed POFA-cement blend concrete. The aim was to determine the place ability and workability properties of the fresh concrete mixture with varying ratios of POFA-cement combinations from Scheffe’s mixture design. The experimental results signify that the slump test value decreases with increasing POFA fractions in the concrete mixture, requiring more water to enhance workability. This may be due to the presence of alumino-silica content in the admixtures and increased surface area. The obtained experimental result is presented in a graphical plot shown in Fig. 4. The obtained results show that Y_1_ experimental point achieved a maximum slump of 125mm with ratio of 0.92:0.44:2.18:3.85:0.03 for cement, water, coarse agg., fine agg., and POFA respectively. In contrast, Y_5_ experimental point achieved a minimum slump of 41mm with mix ratio of 0.5:0.6:1.95:3.72:0.5 for cement, water, coarse agg., fine agg., and POFA respectively. The findings indicate a linear decrease in slump response as the admixture percentages in the mix increase. In the context of this study, the slump test results for palm oil fuel ash (POFA) concrete offer valuable insights into the concrete’s ability to flow, deform, and retain its homogeneity without excessive segregation or bleeding^68^

### Test on the hardened concrete samples

#### Compressive strength Results

Concrete compressive strength results are a vital indicator of the material's ability to withstand compressive loads without failure. It is a fundamental property used to assess the structural integrity and performance of concrete in various engineering applications. To optimize the compressive strength of POFA blended concrete, laboratory responses for compressive strength were collected for fifteen different mixture designs, each with three replicates after 28 days of curing. These values were used to develop the Scheffe's model. Among the results, Y_4_ had the highest compressive strength at 31.16 N/mm^2^, while Y_5_ had the lowest at 19.82 N/mm^2^ (Table 5). Control points were also tested for model validation, with C_35_ showing the highest compressive strength at 28.18 N/mm^2^, and C_45_ having the lowest at 21.82 N/mm^2^ (Table 6). Overall, the incorporation of POFA admixtures in the concrete matrix led to improved mechanical strength, making it suitable for sustainable structural applications^69,70^.

**Table 5:** Compressive strength results.

Actual						Pseudo				
Z1	Z2	Z3	Z4	Z5	Response	X1	X2	X3	X4	X5
0.97	0.44	2.18	3.85	0.03	23.35	1	0	0	0	0
0.88	0.47	1.89	4.15	0.12	25.69	0	1	0	0	0
0.74	0.5	2.23	4	0.26	28.32	0	0	1	0	0
0.65	0.54	2.3	3.96	0.35	31.16	0	0	0	1	0
0.5	0.6	1.95	3.72	0.5	19.82	0	0	0	0	1
0.925	0.455	2.035	4	0.075	21.98	0.5	0.5	0	0	0
0.855	0.47	2.205	3.925	0.145	20.77	0.5	0	0.5	0	0
0.81	0.49	2.24	3.905	0.19	23.56	0.5	0	0	0.5	0
0.735	0.52	2.065	3.785	0.265	26.12	0.5	0	0	0	0.5
0.81	0.485	2.06	4.075	0.19	24.64	0	0.5	0.5	0	0
0.765	0.505	2.095	4.055	0.235	25.25	0	0.5	0	0.5	0
0.69	0.535	1.92	3.935	0.31	27.07	0	0.5	0	0	0.5
0.695	0.52	2.265	3.98	0.305	26.31	0	0	0.5	0.5	0
0.62	0.55	2.09	3.86	0.38	29.28	0	0	0.5	0	0.5
0.575	0.57	2.125	3.84	0.425	28.35	0	0	0	0.5	0.5

**Table 6:** Compressive strength results for control points.

Actual						Pseudo				
Z1	Z2	Z3	Z4	Z5	Response	X1	X2	X3	X4	X5
0.827	0.483	2.085	3.972	0.173	22.99	0.3	0.3	0.3	0	0.1
0.8	0.495	2.106	3.96	0.2	26.21	0.3	0.3	0	0.3	0.1
0.758	0.504	2.208	3.915	0.242	24.80	0.3	0	0.3	0.3	0.1
0.731	0.513	2.121	4.005	0.269	25.46	0	0.3	0.3	0.3	0.1
0.664	0.536	2.162	3.889	0.336	27.17	0.1	0	0.3	0.3	0.3
0.706	0.527	2.06	3.934	0.294	25.32	0.1	0.3	0	0.3	0.3
0.733	0.515	2.039	3.946	0.267	24.63	0.1	0.3	0.3	0	0.3
0.778	0.497	2.144	4.018	0.222	23.88	0.1	0.3	0.3	0.3	0
0.81	0.4875	2.15	3.99	0.19	24.55	0.25	0.25	0.25	0.25	0
0.7725	0.5025	2.0625	3.93	0.2275	25.09	0.25	0.25	0.25	0	0.25
0.75	0.5125	2.08	3.92	0.25	26.24	0.25	0.25	0	0.25	0.25
0.715	0.52	2.165	3.8825	0.285	27.36	0.25	0	0.25	0.25	0.25
0.6925	0.5275	2.0925	3.9575	0.3075	23.61	0	0.25	0.25	0.25	0.25
0.748	0.51	2.11	3.936	0.252	28.18	0.2	0.2	0.2	0.2	0.2
0.842	0.477	2.12	3.996	0.158	21.82	0.3	0.3	0.3	0.1	0

#### Flexural strength response

Concrete flexural strength results provide valuable insights into the material's ability to resist bending or deflection when subjected to external loads. It is a critical mechanical property that reflects the concrete's performance in applications where it needs to span over supports or resist bending stresses, such as in beams, slabs, and other structural elements. Flexural strength testing involves applying a load perpendicular to the surface of a concrete beam or sample until it reaches failure^61^. The flexural strength laboratory responses were obtained for concrete beam samples of dimension 100*100*400 mm cured for 28 days, involving fifteen different mixture design points, each with three replicates. These values were used to develop Scheffe’s second-order regression model for optimizing concrete with POFA blended concrete’s flexural strength properties. Table 7 shows Y_4_ had the maximum strength value at 8.84 N/mm^2^, while Y_5_ and Y_45_ had the minimum at 4.25 and 4.89 N/mm^2^ respectively. Table 8 presents control point responses for validating the regression model, where C_5_ had the highest flexural strength at 7.91 N/mm^2^, and C_45_ had the lowest at 4.96 N/mm^2^. These results demonstrate the improved strength performance of the concrete as POFA percentage replacement of cement in the matrix increases to about 35%^71^.

**Table 7:** Flexural strength experimental response.

Actual						Pseudo				
Z1	Z2	Z3	Z4	Z5	Response	X1	X2	X3	X4	X5
0.97	0.44	2.18	3.85	0.03	5.01	1	0	0	0	0
0.88	0.47	1.89	4.15	0.12	5.83	0	1	0	0	0
0.74	0.5	2.23	4	0.26	6.72	0	0	1	0	0
0.65	0.54	2.3	3.96	0.35	8.84	0	0	0	1	0
0.5	0.6	1.95	3.72	0.5	4.25	0	0	0	0	1
0.925	0.455	2.035	4	0.075	5.26	0.5	0.5	0	0	0
0.855	0.47	2.205	3.925	0.145	5.90	0.5	0	0.5	0	0
0.81	0.49	2.24	3.905	0.19	6.11	0.5	0	0	0.5	0
0.735	0.52	2.065	3.785	0.265	7.07	0.5	0	0	0	0.5
0.81	0.485	2.06	4.075	0.19	6.22	0	0.5	0.5	0	0
0.765	0.505	2.095	4.055	0.235	6.39	0	0.5	0	0.5	0
0.69	0.535	1.92	3.935	0.31	8.14	0	0.5	0	0	0.5
0.695	0.52	2.265	3.98	0.305	7.38	0	0	0.5	0.5	0
0.62	0.55	2.09	3.86	0.38	5.34	0	0	0.5	0	0.5
0.575	0.57	2.125	3.84	0.425	4.89	0	0	0	0.5	0.5

**Table 8:** Flexural strength experimental response for control points.

Actual						Pseudo				
Z1	Z2	Z3	Z4	Z5	Response	X1	X2	X3	X4	X5
0.827	0.483	2.085	3.972	0.173	5.48	0.3	0.3	0.3	0	0.1
0.8	0.495	2.106	3.96	0.2	6.16	0.3	0.3	0	0.3	0.1
0.758	0.504	2.208	3.915	0.242	6.34	0.3	0	0.3	0.3	0.1
0.731	0.513	2.121	4.005	0.269	6.63	0	0.3	0.3	0.3	0.1
0.664	0.536	2.162	3.889	0.336	7.91	0.1	0	0.3	0.3	0.3
0.706	0.527	2.06	3.934	0.294	6.89	0.1	0.3	0	0.3	0.3
0.733	0.515	2.039	3.946	0.267	6.72	0.1	0.3	0.3	0	0.3
0.778	0.497	2.144	4.018	0.222	6.05	0.1	0.3	0.3	0.3	0
0.81	0.4875	2.15	3.99	0.19	5.78	0.25	0.25	0.25	0.25	0
0.7725	0.5025	2.0625	3.93	0.2275	6.26	0.25	0.25	0.25	0	0.25
0.75	0.5125	2.08	3.92	0.25	6.57	0.25	0.25	0	0.25	0.25
0.715	0.52	2.165	3.8825	0.285	7.23	0.25	0	0.25	0.25	0.25
0.6925	0.5275	2.0925	3.9575	0.3075	7.34	0	0.25	0.25	0.25	0.25
0.748	0.51	2.11	3.936	0.252	6.51	0.2	0.2	0.2	0.2	0.2
0.842	0.477	2.12	3.996	0.158	4.96	0.3	0.3	0.3	0.1	0

#### Spitting tensile strength response

Concrete splitting tensile strength results provide important information about the material's ability to resist tensile stresses perpendicular to the applied load. It is a critical mechanical property used to assess the performance and durability of concrete in applications where tensile forces are present, such as in pavements and slabs. The splitting tensile strength results were obtained for cylindrical concrete samples measuring 150 mm in diameter and 300 mm in height. The samples were cured for 28 days, and the testing involved fifteen Scheffe's experimental design points, each with three replicates. The experimental response is shown in Table 9 which presents the highest strength value of 5.23 N/mm^2^ for Y_35_, while Y_1_ had the lowest at 2.08 N/mm^2^. Moreover, Table 10 shows the control point responses for validating the regression model, with C_5_ having the highest strength result of 5.06 N/mm^2^ and C_1_ with the lowest at 3.85 N/mm^2^. These findings indicate that the concrete's strength improves as the percentage of POFA replacement of cement in the matrix increases^45,72^.

**Table 9:** Splitting tensile strength experimental response.

Actual						Pseudo				
Z1	Z2	Z3	Z4	Z5	Response	X1	X2	X3	X4	X5
0.97	0.44	2.18	3.85	0.03	2.08	1	0	0	0	0
0.88	0.47	1.89	4.15	0.12	3.65	0	1	0	0	0
0.74	0.5	2.23	4	0.26	4.49	0	0	1	0	0
0.65	0.54	2.3	3.96	0.35	5.17	0	0	0	1	0
0.5	0.6	1.95	3.72	0.5	3.24	0	0	0	0	1
0.925	0.455	2.035	4	0.075	2.56	0.5	0.5	0	0	0
0.855	0.47	2.205	3.925	0.145	3.70	0.5	0	0.5	0	0
0.81	0.49	2.24	3.905	0.19	3.89	0.5	0	0	0.5	0
0.735	0.52	2.065	3.785	0.265	4.58	0.5	0	0	0	0.5
0.81	0.485	2.06	4.075	0.19	4.01	0	0.5	0.5	0	0
0.765	0.505	2.095	4.055	0.235	4.35	0	0.5	0	0.5	0
0.69	0.535	1.92	3.935	0.31	4.92	0	0.5	0	0	0.5
0.695	0.52	2.265	3.98	0.305	4.74	0	0	0.5	0.5	0
0.62	0.55	2.09	3.86	0.38	5.23	0	0	0.5	0	0.5
0.575	0.57	2.125	3.84	0.425	4.17	0	0	0	0.5	0.5

**Table 10:** Splitting tensile strength experimental response for control points.

Actual						Pseudo				
Z1	Z2	Z3	Z4	Z5	Response	X1	X2	X3	X4	X5
0.827	0.483	2.085	3.972	0.173	3.85	0.3	0.3	0.3	0	0.1
0.8	0.495	2.106	3.96	0.2	3.93	0.3	0.3	0	0.3	0.1
0.758	0.504	2.208	3.915	0.242	4.31	0.3	0	0.3	0.3	0.1
0.731	0.513	2.121	4.005	0.269	4.38	0	0.3	0.3	0.3	0.1
0.664	0.536	2.162	3.889	0.336	5.06	0.1	0	0.3	0.3	0.3
0.706	0.527	2.06	3.934	0.294	4.75	0.1	0.3	0	0.3	0.3
0.733	0.515	2.039	3.946	0.267	4.52	0.1	0.3	0.3	0	0.3
0.778	0.497	2.144	4.018	0.222	4.24	0.1	0.3	0.3	0.3	0
0.81	0.4875	2.15	3.99	0.19	3.88	0.25	0.25	0.25	0.25	0
0.7725	0.5025	2.0625	3.93	0.2275	4.29	0.25	0.25	0.25	0	0.25
0.75	0.5125	2.08	3.92	0.25	4.47	0.25	0.25	0	0.25	0.25
0.715	0.52	2.165	3.8825	0.285	4.71	0.25	0	0.25	0.25	0.25
0.6925	0.5275	2.0925	3.9575	0.3075	5.03	0	0.25	0.25	0.25	0.25
0.748	0.51	2.11	3.936	0.252	4.46	0.2	0.2	0.2	0.2	0.2
0.842	0.477	2.12	3.996	0.158	3.90	0.3	0.3	0.3	0.1	0

### Scheffe’s regression equation

#### Compressive strength

The model equation is derived by inputting the derived experimental response values into Eq. 14, which represents the relationship between the obtained response and the model coefficients, resulting in the Scheffe's coefficients. These coefficient values are then substituted into Eq. (15), leading to the model equation presented in Eq. (20) and Table 11.

20 $$\begin{aligned} {\hat{\text{Y}}}comp & = {23}.{\text{35X}}_{{1}} + { 25}.{\text{69X}}_{{2}} + { 28}.{\text{32X}}_{{3}} + { 31}.{\text{16X}}_{{4}} \\ & \;\; + { 19}.{\text{82X}}_{{5}} {-}{ 1}0.{\text{16X}}_{{1}} {\text{X}}_{{2}} {-}{ 2}0.{\text{26X}}_{{1}} {\text{X}}_{{3}} {-}{ 14}.{\text{78X}}_{{1}} {\text{X}}_{{4}} \\ & \;\; + { 18}.{\text{14X}}_{{1}} {\text{X}}_{{5}} - { 9}.{\text{46X}}_{{2}} {\text{X}}_{{3}} - { 12}.{7}0{\text{X}}_{{2}} {\text{X}}_{{4}} + { 17}.{\text{26X}}_{{2}} {\text{X}}_{{5}} \\ & \;\;{-}{ 13}.{\text{72X}}_{{3}} {\text{X}}_{{4}} + { 2}0.{\text{84X}}_{{3}} {\text{X}}_{{5}} + { 11}.{\text{44X}}_{{4}} {\text{X}}_{{5}} \\ \end{aligned}$$

**Table 11:** Regression model coefficient for compressive strength.

β1	β2	β3	β4	β5	β12	β13	β14	β15	β23	β24	β25	β34	β35	β45
23.35	25.69	28.32	31.16	19.82	− 10.16	− 20.26	− 14.78	18.14	− 9.46	− 12.70	17.26	− 13.72	20.84	11.44

#### Flexural strength model equation

The regression model equation is obtained by substituting the laboratory responses for the concrete's flexural strength into Eq. (15), resulting in Eq. (21) and the coefficients is shown in Table 12

21 $$\begin{aligned} {\hat{\text{Y}}}flex & = { 5}.0{\text{1X}}_{{1}} + { 5}.{\text{83X}}_{{2}} + { 6}.{\text{72X}}_{{3}} + { 8}.{\text{84X}}_{{4}} + { 4}.{\text{25X}}_{{5}} \\ & \;\;\;{-} \, 0.{\text{64X}}_{{1}} {\text{X}}_{{2}} + \, 0.{\text{14X}}_{{1}} {\text{X}}_{{3}} {-}{ 3}.{\text{26X}}_{{1}} {\text{X}}_{{4}} + { 9}.{\text{76X}}_{{1}} {\text{X}}_{{5}} \\ & \;\;\;{-} \, 0.{\text{22X}}_{{2}} {\text{X}}_{{3}} {-}{ 3}.{\text{78X}}_{{2}} {\text{X}}_{{4}} + { 12}.{4}0{\text{X}}_{{2}} {\text{X}}_{{5}} {-}{ 1}.{6}0{\text{X}}_{{3}} {\text{X}}_{{4}} \\ & \;\;\;{-} \, 0.{\text{58X}}_{{3}} {\text{X}}_{{5}} {-}{ 6}.{\text{62X}}_{{4}} {\text{X}}_{{5}} \\ \end{aligned}$$

**Table 12:** Regression model coefficient for flexural strength.

β1	β2	β3	β4	β5	β12	β13	β14	β15	β23	β24	β25	β34	β35	β45
5.01	5.83	6.72	8.84	4.25	− 0.64	0.14	− 3.26	9.76	− 0.22	− 3.78	12.40	− 1.60	− 0.58	− 6.62

#### Splitting tensile strength model equation

The Scheffe’s quadratic model equation is derived by incorporating the experimental results for the concrete's splitting tensile strength into Eq. (15), which leads to the formation of Eq. (22). The regression coefficients obtained from this process are presented in Table 13.

22 $$\begin{aligned} {\hat{\text{Y}}}split & = { 2}.0{\text{8X}}_{{1}} + { 3}.{\text{65X}}_{{2}} + { 4}.{\text{49X}}_{{3}} + { 5}.{\text{17X}}_{{4}} + { 3}.{\text{24X}}_{{5}} \\ & \;\;\;{-}{ 1}.{\text{22X}}_{{1}} {\text{X}}_{{2}} + { 1}.{\text{66X}}_{{1}} {\text{X}}_{{3}} + { 1}.0{\text{6X}}_{{1}} {\text{X}}_{{4}} + { 7}.{\text{68X}}_{{1}} {\text{X}}_{{5}} {-} \, 0.{\text{24X}}_{{2}} {\text{X}}_{{3}} \\ & \;\;\;{-} \, 0.{\text{24X}}_{{2}} {\text{X}}_{{4}} + { 5}.{9}0{\text{X}}_{{2}} {\text{X}}_{{5}} {-} \, 0.{\text{36X}}_{{3}} {\text{X}}_{{4}} + { 5}.{\text{468X}}_{{3}} {\text{X}}_{{5}} {-} \, 0.{\text{14X}}_{{4}} {\text{X}}_{{5}} \\ \end{aligned}$$

**Table 13:** Regression model coefficient for flexural strength.

β1	β2	β3	β4	β5	β12	β13	β14	β15	β23	β24	β25	β34	β35	β45
2.08	3.65	4.49	5.17	3.24	− 1.22	1.66	1.06	7.68	− 0.24	− 0.24	5.90	− 0.36	5.46	− 0.14

### Test of adequacy and validation of developed models

**Figure 5:**
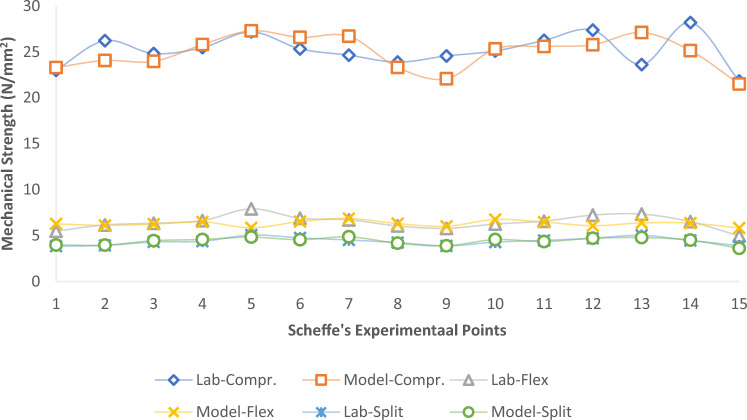
Comparison of the developed model and experimental results.

Regression model validation is a crucial step in the analysis of data and the development of predictive models. In the context of a regression model, validation refers to the process of assessing the model's accuracy and reliability in predicting outcomes for new or unseen data. This helps in gauging the model's generalization capability, which is essential for its practical applicability^73^. The control points of the experiment were employed to assess the accuracy of the developed Scheffe's model using statistical assessment method such as analysis of variance (ANOVA). Graphical representations in Fig. 5 display a comparison between the actual experimental results and the values obtained from the generated regression second-order model for the compressive, flexural and splitting tensile strength properties^74^.

#### Statistical evaluation of model performance using ANOVA

Statistical evaluation of model performance using ANOVA is a powerful technique commonly used to assess the accuracy and significance of regression models. ANOVA helps to determine whether the variation in the response variable can be adequately explained by the model's predictors. It essentially compares the variability of the observed data to the variability of the data predicted by the regression model. The main objective is to evaluate whether the model's predictors (independent variables) have a significant impact on the response variable (dependent variable)^75^.

A significant ANOVA result indicates that the regression model is valid and provides valuable insights into the relationship between the predictors and the response variable. However, a non-significant ANOVA result suggests that the model might not be appropriate, and further investigation or model refinement may be necessary. In summary, ANOVA is a fundamental tool for statistical evaluation in regression analysis, helping researchers and analysts determine the adequacy and significance of their models, ultimately leading to better decision-making and insights from the data^76^.

Analysis of variance (ANOVA) was carried out using Microsoft Excel statistical software which was deployed to evaluate the prediction performance of the developed second-order regression models for the compressive, flexural and splitting tensile strength optimization. This evaluation is based on the comparison of laboratory and model-predicted values at a 95% confidence interval, as presented in Tables 14, 15 and 16. The condition for assessment is as follows: if F > F crit, we reject the null hypothesis. After performing the statistical computation, for the compressive strength we find that F = 0.165 and F crit = 4.196. As F crit > F, we accept the null hypothesis with a p-value of 0.688, which is greater than the alpha value of 0.05^77,78^.

**Table 14:** ANOVA results for compressive strength.

Groups	Count	Sum	Average	Variance		
lab-comp	15	377.31	25.154	2.934097		
model-comp	15	373.4055	24.8937	3.226286		

Source of variation	SS	df	MS	F	P-value	F crit
Between groups	0.508171	1	0.508171	0.16498	0.6877	4.195972
Within groups	86.24536	28	3.080191			
Total	86.75353	29

**Table 15:** ANOVA results for flexural strength.

Groups	Count	Sum	Average	Variance		
lab-flex	15	96.83	6.455333	0.553627		
model-flex	15	94.5516	6.30344	0.089781		

Source of variation	SS	df	MS	F	P-value	F crit
Between groups	0.173037	1	0.173037	0.537876	0.469411	4.195972
Within groups	9.007713	28	0.321704			
Total	9.18075	29

**Table 16:** ANOVA results for splitting tensile strength.

Groups	Count	Sum	Average	Variance		
lab-split	15	65.78	4.385333	0.154341		
model-split	15	65.8091	4.387273	0.146169		

Source of variation	SS	df	MS	F	P-value	F crit
Between groups	2.82E−05	1	2.82E−05	0.000188	0.989162	4.195972
Within groups	4.207138	28	0.150255			
Total	4.207166	29				

Similarly, for the flexural strength we obtained F = 0.538, F crit = 4.196 and p-value of 0.47. Also, we observe the criteria that F crit > F. finally, for the splitting tensile strength results, F = 0.000188, F crit = 4.196 and p-value of 0.989 was calculated which indicated that F crit > F. The statistical examination outcome showed that the difference between the actual laboratory-derived results and the model results was not significant which signified that the models developed possessed good prediction accuracy^79,80^.

## Conclusion

The study focused on exploring the mechanical behaviour of green concrete containing palm oil fuel ash (POFA) through an optimization process using Scheffe's theory. The conclusions drawn from the research findings and results are as follows:

1. The study employs Scheffe's quadratic polynomial to predict mechanical properties in POFA-cement concrete. The model estimates strength values based on mix ratios or determines ratios for desired strengths.
2. The evaluation of the rheological properties of the freshly mixed POFA-cement blended concrete shows that the incorporation of admixtures led to a reduction in the slump response of the fresh blended concrete specimens.
3. The maximum compressive and flexural strength at 28 days were 31.16 N/mm^2^ and 8.84 N/mm^2^, achieved with a mix of 0.65:0.54:2.3:3.96:0.35 for cement, water, coarse aggregate, Fine aggregate and POFA respectively. Additionally, splitting tensile strength reached 8.84 N/mm^2^ with a mix of 0.62:0.55:2.09:3.86:0.38.
4. The lowest compressive and flexural strength, at 19.82 N/mm^2^ and 4.25 N/mm^2^, was achieved with a mix of 0.5:0.6:0.95:3.72:0.5 for cement, water, coarse aggregate, Fine aggregate and POFA respectively. Similarly, splitting tensile strength was minimal at 2.08 N/mm^2^ with a mix of 0.97:0.44:2.18:3.85:0.03.
5. The model's accuracy was assessed through ANOVA, revealing a strong correlation between the model's predictions and the laboratory-derived control results. The strengths predicted by the model closely align with the corresponding experimentally observed outcomes, indicating good agreement between them.
6. Furthermore, using desired percentages of agricultural waste and their derivatives such as POFA in concrete production leads to cost savings, promoting sustainability, and playing a significant role in waste management.

### Research limitations

1. Limited Scope of Variables: One limitation of this research is the focus on specific variables for optimization, such as mix proportions and curing conditions.
2. Short-Term Evaluation: The mechanical properties of concrete were evaluated after 28 days of curing. Longer-term studies tracking the performance over several years or decades would provide a more comprehensive understanding of how POFA concrete behaves over time.
3. Laboratory Setting: The research was conducted in a controlled laboratory environment. Real-world construction sites may have variations in conditions that could affect the performance of POFA concrete differently. Field studies and on-site assessments could complement these findings.
4. Material Variability: The properties of POFA can vary based on its source and processing methods. Future research should explore how these variations impact the effectiveness of Scheffe's optimization in different contexts.

### Recommendations for future studies

Although the current research on Scheffe's optimization of POFA concrete offers valuable insights, there are opportunities for future work to address limitations and further advance the understanding and practical implementation of this sustainable construction material. Future research on the optimization of POFA concrete using Scheffe's method should encompass a broader range of variables, emphasize long-term performance, consider environmental and economic aspects, comprehensive life cycle assessments and focus on practical implementation in real-world construction projects. These recommendations can contribute to the sustainable advancement of the construction industry.

## Data Availability

The datasets generated and analyzed during the current study are available from the corresponding author on reasonable request.
